# Nitrogen starvation-induced transcriptome alterations and influence of transcription regulator mutants in *Mycobacterium smegmatis*

**DOI:** 10.1186/1756-0500-6-482

**Published:** 2013-11-22

**Authors:** Nadja Jeßberger, Yinhua Lu, Johannes Amon, Fritz Titgemeyer, Sophia Sonnewald, Stephen Reid, Andreas Burkovski

**Affiliations:** 1 Lehrstuhl für Mikrobiologie, Friedrich-Alexander-Universität Erlangen-Nürnberg, Erlangen, Germany; 2 Key Laboratory of Synthetic Biology, Institute of Plant Physiology and Ecology, Shanghai Institutes for Biological Sciences, Chinese Academy of Sciences, Shanghai, People’s Republic of China; 3 Fachbereich Oecotrophologie, Fachhochschule Münster, Münster, Germany; 4 Lehrstuhl für Biochemie, Friedrich-Alexander-Universität Erlangen-Nürnberg, Erlangen, Germany

**Keywords:** AmtR, GlnR, Nitrogen control, Nitrogen metabolism, OmpR/EnvZ

## Abstract

**Background:**

As other bacteria, *Mycobacterium smegmatis* needs adaption mechanisms to cope with changing nitrogen sources and to survive situations of nitrogen starvation. In the study presented here, transcriptome analyses were used to characterize the response of the bacterium to nitrogen starvation and to elucidate the role of specific transcriptional regulators.

**Results:**

In response to nitrogen deprivation, a general starvation response is induced in *M. smegmatis*. This includes changes in the transcription of several hundred genes encoding e.g. transport proteins, proteins involved in nitrogen metabolism and regulation, energy generation and protein turnover. The specific nitrogen-related changes at the transcriptional level depend mainly on the presence of GlnR, while the AmtR protein controls only a small number of genes.

**Conclusions:**

*M. smegmatis* is able to metabolize a number of different nitrogen sources and nitrogen control in *M. smegmatis* is similar to control mechanisms characterized in streptomycetes, while the master regulator of nitrogen control in corynebacteria, AmtR, is plays a minor role in this regulatory network.

## Background

Nitrogen is one of the macro-elements of life and constituent of numerous cellular metabolites (e.g. amino acids, amino sugars, nucleotides) and macro-molecules (proteins, peptidoglycan, DNA, mRNA). Therefore, its supply is crucial for organisms. Bacteria are able to use a number of different nitrogen sources to cover their nitrogen requirements; however, in most species metabolism is biased in favor of ammonium. When ammonium becomes limiting, typical bacterial responses to this situation include the expression and activation of ammonium carriers and high affinity ammonium assimilation enzymes as well as expression of carriers and enzymes for the use of alternative nitrogen sources. This response, designated as nitrogen control, is governed by different types of transcriptional regulators. In respect to these transcription control proteins, *Actinobacteria* are divided into two major groups, one comprising GlnR and the other AmtR as master regulators of nitrogen control. GlnR was described for example in *Streptomyces coelicolor*, *Streptomyces venezuelae*, *Amycolatopsis mediterranei* and *Mycobacterium tuberculosis*, while AmtR controls nitrogen regulation in corynebacteria such as *Corynebacterium diphtheriae*, *Corynebacterium efficiens* and *Corynebacterium glutamicum*[1]*.*

In *S. coelicolor* the OmpR-type regulator GlnR acts as a transcriptional activator for at least 15 genes, encoding proteins related to nitrogen uptake, metabolism and regulation as well as proteins with unknown function [2,3]. Proteomics analyses indicated broader regulatory influence, e.g. on amino acid and carbon metabolism [4]. *S. coelicolor* exhibits a second GlnR-type regulator, designated GlnRII, which was shown to bind to the upstream regions of the *amtB-glnK-glnD* operons and the *glnA* and *glnII* gene [2,3,5]. The regulatory function of this protein is still unknown.

In corynebacteria, like the amino acid producing strains *C. glutamicum* and *C. efficiens* or the pathogen *C. diphtheriae*, expression of genes coding for proteins for uptake and assimilation of nitrogen sources is under control of the TetR-type regulator AmtR [6-8]. In *C. glutamicum* AmtR blocks transcription of at least 35 genes during sufficient ammonium supply [9,10]. The corresponding regulon comprises the *amtA* and *amtB* genes, which encode ammonium transporters, as well as *gdh*, *glnA* and *gltBD*, which code for enzymes crucial for ammonium assimilation, i.e. glutamate dehydrogenase, glutamine synthetase and glutamate synthase. Furthermore, transcription of the *crnT* and *codA* gene, responsible for creatinine transport and metabolism as well as the *urtABCDE* and *ureABCEFGD* operon, encoding a urea ABC transporter and urease, are controlled by AmtR in addition to a number of genes coding for biochemically uncharacterized enzymes and transport systems as well as signal transduction proteins.

In *M. smegmatis* transcription of *amt1,* encoding a putative ammonium permease, the *amtB-glnK-glnD* operon, which codes for an additional ammonium transporter and signal transduction proteins, as well as *glnA*, coding for glutamine synthetase, are positively controlled by GlnR [11]. Besides these genes encoding proteins for ammonium uptake and assimilation, the regulation of other genes and operons encoding various (putative) transporters of alternative nitrogen sources, e.g. *narK*, *narK3*, *urtABCDE*, as well as nitrogen assimilatory enzymes, e.g. *gdh*, *glnA2*, *glnA3*, *glnA4*, *gltBD*, *narIJHG*, *nirBD*, *ureABCFG*, *ureEFABCGD*, was unclear until now (for review, see [1,12]). Furthermore, *M. smegmatis* belongs to a group of *Actinobacteria* including e.g. *Nocardia farcinica*, *Rhodococcus* sp., *Clavibacter michiganensis* and *Kineococcus radiotolerans* that exhibit both, GlnR and AmtR. In none of these species, the function of AmtR and its interaction with GlnR have been addressed until now.

To expand our knowledge of nitrogen starvation response in *M. smegmatis* and to shed light on the function and interplay of GlnR and AmtR in this organism, DNA microarray analyses were carried out, with the aim to characterize the nitrogen control network of *M. smegmatis* in more detail.

## Results and discussion

### Nitrogen starvation-dependent transcription of genes

For a global analysis of nitrogen-starvation-induced genes, wild-type cells were grown up to the mid-exponential growth phase, when nitrogen starvation was induced by washing the cells and transferring them to minimal medium without nitrogen source. Total RNA was isolated after 0.5 hours of starvation and hybridized together with RNA from untreated control cultures to DNA microarrays. As shown by Amon and co-workers [11], this procedure is sufficient for full induction of genes encoding ammonium transporters and high affinity ammonium assimilation enzymes. In response to the induction of nitrogen starvation, transcripts of 231 genes were found to be decreased and of 284 genes to be increased by at least a factor of 3 in the wild-type (Additional file 1: Table S1 and S2). These were classified according to functional categories [13].

**Figure 1:**
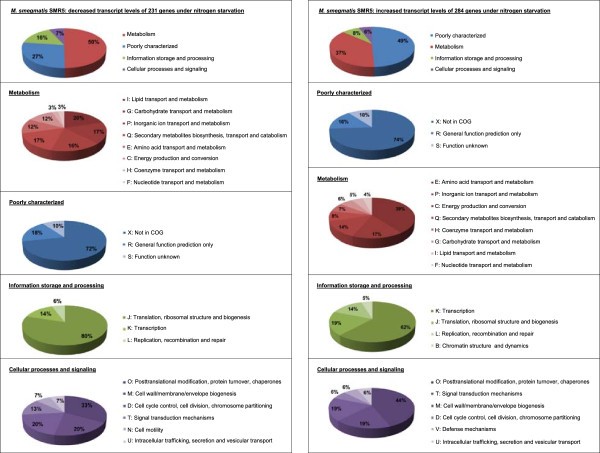
**Functional catagories of the *M. smegmatis *nitrogen starvation stimulon.** DNA microarray analyses were carried out with RNA isolated from nitrogen-supplied and nitrogen-starved wild-type cells. Genes with decreased **(A)** and increased **(B)** transcript levels in response to nitrogen starvation were classified according to functional categories [13].

COG classification of genes with decreased mRNA level (Figure 1A) revealed that 50% of these were allocated to the major group *metabolism*, another 27% to the major category *poorly characterized*. 16% of the genes with decreased transcript amounts in the wild-type under nitrogen starvation belonged to the group *information storage and processing* and 7% correlated with the group *cellular processes and signaling*. Only 4% of these 231 genes are described as putatively involved in nitrogen metabolism.

When the 284 genes with enhanced transcript levels upon starvation were analyzed, 12.5% were associated with nitrogen metabolism. Classification of these genes into COGs (Figure 1B) shows that 49% of these genes were *poorly characterized*, 37% belonged to the major category *metabolism*, 8% of the genes with enhanced transcript amounts in the wild-type under nitrogen starvation were allocated to *information storage and processing*, while the remaining 6% of genes belonged to *cellular processes and signaling*.

All in all, the microarray results indicated a general adaptation of *M. smegmatis* wild-type to the situation of growth arrest induced by nitrogen starvation. This includes not only nitrogen control, but influences other regulatory networks such as carbon and energy metabolism as well and a similar adaptation strategy was also shown previously for the closely related actinomycete *C. glutamicum*[14].

### Transcriptome analyses of wild-type and glnR mutant strain

**Figure 2:**
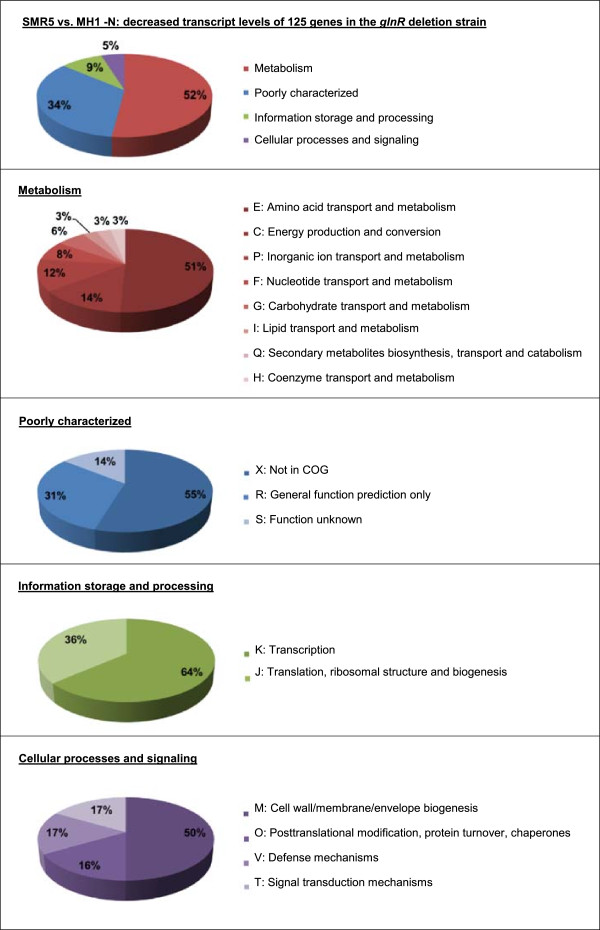
**The GlnR modulon of *M. smegmatis*.** DNA microarray analyses were carried out with RNA isolated from nitrogen-starved wild-type SMR5 and *glnR* deletion strain MH1. Genes with decreased transcript levels in *glnR* deletion strain MH1 were classified according to functional categories [13].

In order to elucidate the specific nitrogen starvation response in more detail, the global transcription patterns of nitrogen-deprived wild-type and *ΔglnR* strain MH1 were compared. Transcripts of 125 genes were up-regulated and of six genes down-regulated by at least a factor of 3 in the wild-type in comparison to MH1, when the two strains were starved for nitrogen. The classification of these genes into COGs (Figure 2) gave a first hint that GlnR is not only involved in the regulation of ammonium assimilation, which was further supported by a detailed inspection of the DNA microarray results (Table 1). Putative GlnR regulated genes included the genes *amtB* (*msmeg_2425*, 56 x), *glnK* (*msmeg_2426*, 53 x), *glnA* (*msmeg_4290*, 34 x), *glnD* (*msmeg_2427*, 18 x) and *amt1* (*msmeg_6259*, 12 x), which were described previously [11] besides others with obvious function in nitrogen metabolism. These include for example the *nirBD* gene cluster (*msmeg_0427-0428*) coding for nitrite reductase subunits, *msmeg_0781*, *msmeg_1052*, *msmeg_2184* and *msmeg_6735*, encoding different putative permeases and transporter subunits involved in amino acid uptake, *msmeg_1293*, *msmeg_2748*, *msmeg_4011*, *msmeg_5730* and *msmeg_6660*, encoding proteins putatively involved in purine and pyrimidine transport, as well as *msmeg_2187*, *msmeg_2981* (*urtB*), *msmeg_2982* (*urtA*) and *msmeg_3626*, encoding proteins for urea uptake and utilization. All in all, the results of transcriptome analyses of nitrogen-deprived wild-type and *ΔglnR* strain MH1 indicated a more global function of GlnR in the regulation of nitrogen metabolism. Similar results were obtained for *S. coelicolor*[3] and together indicate a more general role of GlnR in *Actinobacteria*.

**Table 1:** **Genes with increased mRNA amounts in the wild-type compared to *glnR *** **deletion strain MH1 in response to nitrogen starvation**

**Gene identifier**	**Fold change**	**Annotation**
*msmeg_3358*	3.02	YaeQ protein
*msmeg_4965*	3.07	Hypothetical protein
*msmeg_6507*	3.07	Glycogen debranching enyme GlgX (*glgX*)
*msmeg_1792*	3.12	Conserved hypothetical protein
*msmeg_4567*	3.14	Conserved hypothetical protein
*msmeg_4382*	3.15	Dehydrogenase-reductase SDR family member 10
*msmeg_4011*	3.26	Putative pyrimidine permease RutG
*msmeg_2116*	3.33	PTS system, glucose-specific IIBC component
*msmeg_4570*	3.34	Conserved hypothetical protein
*msmeg_2187*	3.36	Urea amidolyase
*msmeg_4569*	3.48	Conserved hypothetical protein
*msmeg_1292*	3.48	FAD binding domain in molybdopterin dehydrogenase protein
*msmeg_4171*	3.58	Ribose transport system permease protein RbsC
*msmeg_3994*	3.64	Short chain dehydrogenase
*msmeg_5648*	3.68	Hypothetical protein
*msmeg_2189*	3.68	Allophanate hydrolase (*atF*)
*msmeg_0393*	3.73	Fmt protein
*msmeg_1153*	3.73	FAD dependent oxidoreductase
*msmeg_6659*	3.74	Hypothetical protein
*msmeg_6332*	3.79	Amino acid ABC transporter, permease protein
*msmeg_3626*	3.89	Urease, beta subunit (*ureB*)
*msmeg_5083*	4.00	Conserved hypothetical protein
*msmeg_1152*	4.01	Citrate-proton symporter
*msmeg_5331*	4.04	UDP-glucoronosyl and UDP-glucosyl transferase family
*msmeg_1151*	4.08	DNA-binding protein
*msmeg_5783*	4.10	Acetyltransferase, GNAT family
*msmeg_1155*	4.16	Carnitinyl-CoA dehydratase
*msmeg_1157*	4.17	Short chain dehydrogenase
*msmeg_3912*	4.20	Acetoacetyl-CoA reductase
*msmeg_0565*	4.33	Putative glycosyl transferases group 1
*msmeg_1184*	4.40	Serine esterase, cutinase family
*msmeg_1156*	4.44	Dihydrodipicolinate synthetase
*msmeg_1185*	4.46	Transcriptional regulator, AsnC family
*msmeg_1089*	4.61	Hypothetical protein
*msmeg_1088*	4.63	Glutamyl-tRNA(Gln)-aspartyl-tRNA(Asn) amidotransferase
*msmeg_0505*	4.72	Probable sugar ABC transporter, substrate-binding protein
*msmeg_6880*	4.92	Hydrophobic amino acid ABC transporter, putative
*msmeg_6879*	4.97	Nat permease for neutral amino acids NatD
*msmeg_3722*	5.04	Bifunctional coenyme PQQ synthesis protein C-D
*msmeg_1596*	5.05	Transcriptional regulator
*msmeg_6264*	5.06	Putative oxidoreductase
*msmeg_2523*	5.11	Efflux ABC transporter, permease protein, putative
*msmeg_4381*	5.14	Amidase
*msmeg_1090*	5.24	Amidase
*msmeg_1508*	5.54	Amino acid permease-associated region
*msmeg_5729*	5.75	Hydantoin racemase
*msmeg_6733*	6.32	Hydrolase, carbon-nitrogen family
*msmeg_2748*	6.36	Soluble pyridine nucleotide transhydrogenase (*sthA*)
*msmeg_1085*	6.41	Dipeptide transport system permease protein DppB
*msmeg_2569*	6.61	Oxidoreductase, 2OG-Fe(II) oxygenase family
*msmeg_0429*	6.64	Putative ferric uptake regulator
*msmeg_6878*	6.97	Inner-membrane translocator
*msmeg_6263*	7.01	Glutamate synthase family protein
*msmeg_6262*	7.40	FwdC-FmdC family protein
*msmeg_1295*	7.42	Transthyretin
*msmeg_3402*	7.82	Cytosine permease, putative
*msmeg_5356*	8.15	Hypothetical protein
*msmeg_1296*	8.73	Uricase
*msmeg_0780*	8.79	Phosphotransferase enyme family protein
*msmeg_6877*	8.90	Branched-chain amino acid transport system ATP-binding protein
*msmeg_0566*	8.94	Aliphatic amidase
*msmeg_3403*	9.26	Formamidase
*msmeg_1990*	9.45	Conserved hypothetical protein
*msmeg_1086*	9.94	ABC transporter permease protein
*msmeg_0778*	10.22	Putative transcriptional regulator
*msmeg_6261*	10.36	Glutamine amidotransferase, class II
*msmeg_6660*	10.75	Permease, cytosine-purines, uracil, thiamine, allantoin family
*msmeg_6817*	11.34	RNA polymerase sigma factor, sigma-70 family
*msmeg_1052*	11.63	Amino acid carrier protein
*msmeg_6259*	11.67	Ammonium transporter (*amt1*)
*msmeg_6881*	11.75	Transcriptional regulator, GntR family
*msmeg_2185*	12.81	Conserved hypothetical protein
*msmeg_2525*	14.19	Amino acid permease superfamily
*msmeg_1293*	14.73	Xanthine-uracil permeases family protein
*msmeg_2978*	15.78	ABC transporter ATP-binding protein
*msmeg_6260*	16.11	Glutamine synthetase, type III (*glnT*)
*msmeg_4206*	16.44	Molybdopterin oxidoreductase
*msmeg_3400*	17.66	Glutamyl-tRNA(Gln) amidotransferase subunit A
*msmeg_0572*	17.72	Conserved hypothetical protein
*msmeg_2427*	18.22	Protein P-II uridylyltransferase (*glnD*)
*msmeg_4637*	18.43	Conserved hypothetical protein
*msmeg_3401*	18.68	LamB-YcsF family protein
*msmeg_1988*	18.75	Conserved hypothetical protein
*msmeg_1597*	18.80	Transcription factor WhiB
*msmeg_2979*	18.88	ABC transporter ATP-binding protein
*msmeg_2981*	19.17	Branched-chain amino acid ABC-type transport system
*msmeg_6115*	19.21	Phosphoglycerate dehydrogenase
*msmeg_6735*	19.26	Amino acid permease, putative
*msmeg_1087*	19.33	Oligopeptide ABC transporter ATP-binding protein
*msmeg_6116*	19.89	Conserved hypothetical protein
*msmeg_5084*	20.59	Glycosyl transferase, group 2 family protein
*msmeg_5359*	21.35	Cyanate hydratase (*cynS*)
*msmeg_5360*	22.01	Formate-nitrate transporter
*msmeg_4501*	22.33	Sodium:dicarboxylate symporter
*msmeg_4635*	22.90	Ammonium transporter family protein
*msmeg_2980*	23.33	Putative membrane protein
*msmeg_2524*	24.34	ABC transporter, ATP-binding protein
*msmeg_2522*	24.38	Efflux ABC transporter, permease protein
*msmeg_5358*	25.10	Acetamidase-Formamidase family
*msmeg_4294*	26.01	Glutamine synthetase, type I (*glnA*)
*msmeg_5765*	29.55	Globin
*msmeg_4638*	30.14	Vanillate O-demethylase oxidoreductase
*msmeg_4636*	31.81	Hypothetical protein
*msmeg_2186*	32.35	Conserved hypothetical protein
*msmeg_0570*	32.58	Conserved hypothetical protein
*msmeg_6816*	33.03	Molybdopterin oxidoreductase
*msmeg_0432*	34.03	Uroporphyrinogen-III synthetase
*msmeg_4290*	34.13	Glutamine synthetase, type I (*glnA*)
*msmeg_0569*	34.95	Flavoprotein involved in K^+^ transport
*msmeg_0779*	35.16	Short-chain dehydrogenase-reductase SDR
*msmeg_0781*	36.20	Amino acid permease
*msmeg_1987*	42.51	Conserved hypothetical protein
*msmeg_5730*	45.31	Permease for cytosine-purines, uracil, thiamine, allantoin
*msmeg_1082*	47.68	Putative response regulator
*msmeg_0571*	48.04	Hydrolase, carbon-nitrogen family
*msmeg_2184*	48.27	Amino acid permease
*msmeg_0428*	48.97	Nitrite reductase [NAD(P)H] small subunit (*nirD*)
*msmeg_2426*	51.56	Nitrogen regulatory protein P-II (*glnK*)
*msmeg_1084*	51.81	Peptide-opine-nickel uptake family ABC transporter
*msmeg_0427*	52.06	Nitrite reductase [NAD(P)H], large subunit (*nirB*)
*msmeg_0433*	54.53	Nitrite extrusion protein
*msmeg_2425*	56.21	Ammonium transporter (*amtB*)
*msmeg_6734*	62.84	Dibenzothiophene desulfurization enzyme A
*msmeg_2526*	66.62	Copper methylamine oxidase
*msmeg_2982*	136.17	Putative periplasmic binding protein (*urtA*)

The genes showing decreased transcript amounts (Table 2) had no obvious connection to nitrogen metabolism and were not further characterized. Four of these are encoding hypothetical proteins, two code for uncharacterized proteins with connection to hydrogenase expression.

**Table 2:** **Genes with decreased mRNA amounts in the wild-type compared to *glnR *** **deletion strain in response to nitrogen starvation**

**Gene identifier**	**Fold change**	**Annotation**
*msmeg_6498*	3.96	Hypothetical protein
*msmeg_2274*	4.24	Hydrogenase assembly chaperone HypC-HupF (*hypC*)
*msmeg_2275*	4.37	Hydrogenase expression-formation protein HypD (*hypD*)
*msmeg_1738*	4.46	Probable conserved transmembrane protein
*msmeg_3680*	4.61	Hypothetical protein
*msmeg_1999*	10.08	Hypothetical protein

### Validation of DNA microarray results

**Figure 3:**
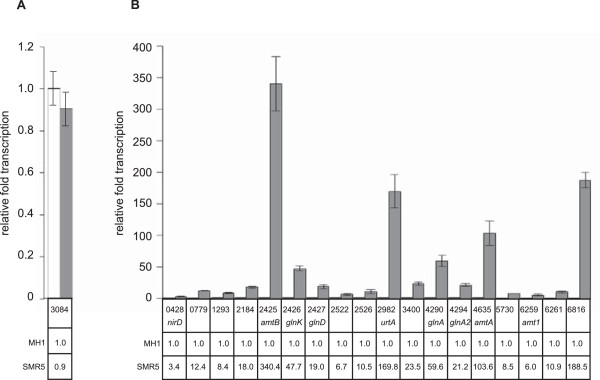
**Verification of DNA affinity microarray results by quantitative RT-PCR.** Total RNA of strains SMR5 and MH1 incubated for 30 min under nitrogen starvation was prepared and used as template for reverse transcription and PCR reaction. Specific primers were used for amplification of 100 bp fragments of target genes. **(A)** The gene *msmeg_3084* was used as control; transcription of this housekeeping gene encoding glyceraldehyde-3-phosphate dehydrogenase was not significantly different in wild-type (grey bar) and *glnR* deletion strain (white bar). **(B)** Relative fold transcription of 20 target genes in the wild-type SMR5 was calculated (grey bars), while the transcription in the *glnR* deletion strain was set one (white bars). Relative fold transcription was calculated in normalization to the reference gene *msmeg_3084*. Genes are sorted according to their msmeg numbers.

The dependence of transcription changes of selected genes on the presence of GlnR was validated by RNA hybridization experiments (data not shown) and quantitative RT-PCR (qPCR; see Figure 3). For qPCR, the transcript of *msmeg_3084* was used as internal control (Figure 3A). An increase of mRNA level in response to nitrogen starvation in the wild-type compared to *glnR* deletion strain MH1 was verified for all targets tested, i.e. *nirD*, *msmeg_0779*, *msmeg_1293*, *msmeg_2184*, the *amtB-glnK-glnD* operon, *msmeg_2522*, *msmeg_2526*, *urtA*, *msmeg_3400*, *glnA*, *glnA2*, *amtA*, *msmeg_5730*, *amt1*, *msmeg_6261* and *msmeg_6816* (Figure 3B).

The real-time RT-PCR experiments validated the DNA microarray analyses; however, from the transcriptome analyses, no conclusion about direct or indirect influences of GlnR can be drawn, since for example changing metabolite concentrations might influence transcription patterns of genes.

### Binding of GlnR upstream of putative target genes

**Figure 4:**
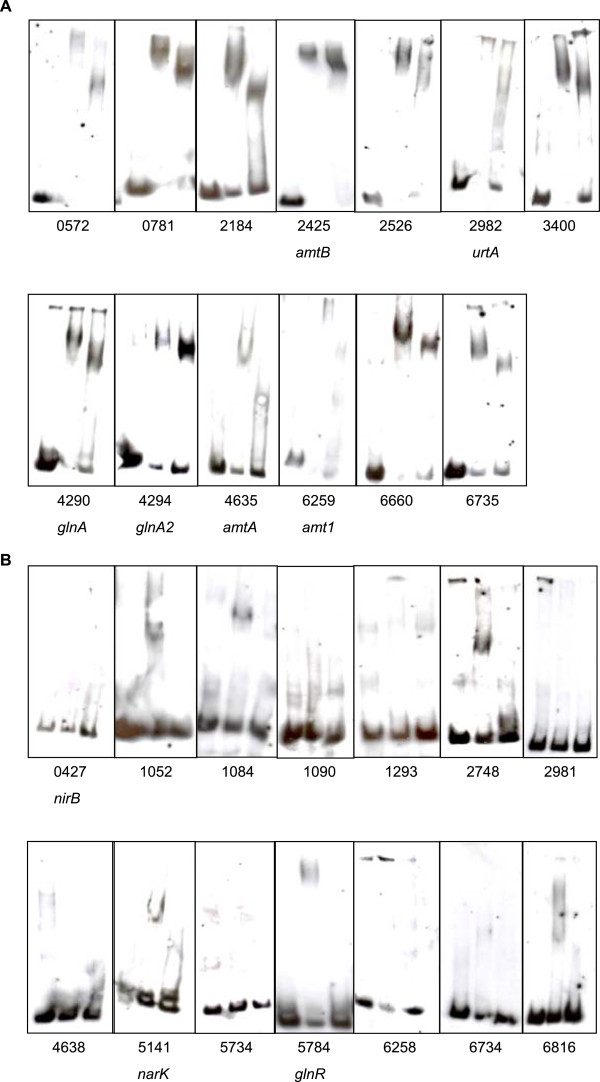
**Test of GlnR binding by gel retardation assays.** 200**–**300 bp DNA fragments upstream of GlnR target genes labeled with digoxigenin were used. For each gene free DNA (lane 1), DNA plus 400 ng MBP-GlnR (lane 2) and DNA plus 400 ng MBP-GlnR plus 3 μg competitor DNA polyd[I-C] (lane 3) was tested. **(A)** Target genes with specific binding; **(B)** unspecific or no binding.

In order to test binding of GlnR to selected putative target DNAs, 200 to 300 bp DNA fragments located upstream of different genes were amplified by PCR and used together with purified MBP-GlnR fusion protein in gel retardation experiments (Figure 4). Specific binding of GlnR to the upstream DNA of *msmeg_0572*, *msmeg_0781*, *msmeg_2184*, *amtB*, *msmeg_2526*, *urtA*, *msmeg_3400*, *glnA*, *glnA2*, *amtA* and *amt1* was shown (Figure 4A).

Binding was not detected for the *nirB, msmeg_1052*, *msmeg_1084*, *msmeg_1090*, *msmeg_1293*, *msmeg_2748*, *msmeg_2981*, *msmeg_4638*, *narK*, *msmeg_5734*, *glnR*, *msmeg_6258*, *msmeg_6734* and *msmeg_6816* upstream region (Figure 4B). In these cases, longer DNA fragments spanning from 300 to 500 bps of the respective upstream region were also tested; however, without positive result (data not shown).

**Figure 5:**
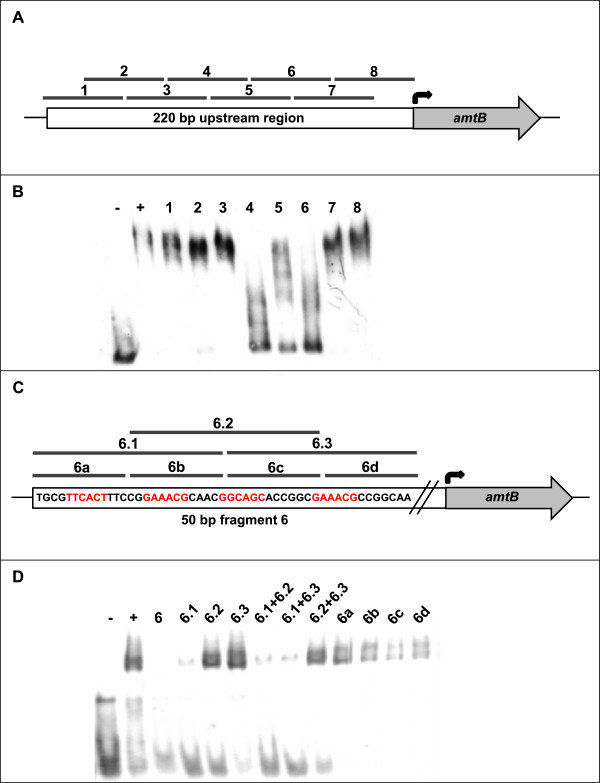
**Competitive gel retardation experiment. (A)** Model of eight overlapping 50 bp DNA fragments covering the 220 bp upstream region of *amtB.***(B)**. When each of these fragments was added in 1000-fold surplus to gel retardation samples consisting of 220 bp digoxigenin-labeled *amtB* promoter fragment and purified MBP-GlnR, an inhibition of the shift was spotted for fragments 4, 5, 6 and weakly 2. **(C)** Model of three overlapping 25 bp DNA fragments (6.1-6.3) and four 15 bp fragments (6a-d) covering the 50 bp fragment 6 in the upstream region of *amtB*. **(D)** Addition of these fragments in 1,000-fold surplus to gel retardation samples containing 50 bp digoxigenin-labeled fragment 6 and 600 ng MBP-GlnR each. An inhibition of the shift was detected for fragment 6.1. -: free DNA as negative control. +: DNA plus 600 ng MBP-GlnR as positive control.

To validate GlnR binding and to localize the GlnR binding site in more detail, a 220 bp fragment upstream of *amtB* was chosen and competitive gel retardation assays were performed (Figure 5). When 50 bp overlapping DNA fragments covering the whole promoter sequence were added, fragments 1, 3, 7 and 8 did not lead to any inhibition of the DNA shift caused by GlnR binding. Addition of fragments 4, 5 and 6 led to strong inhibition, indicating that the GlnR binding site is located in this 100 bp fragment. An additional weak binding site might exist in fragment 2, as a very slight inhibition was also spotted here (Figure 5A and B).

To further specify the localization, the 50 bp fragments 4 and 6 which caused inhibition of GlnR-binding to the 220 bp promoter fragment were used as digoxigenin-labeled DNA probes and 25 as well as 15 bp DNA fragments were used as competitor DNA. While no inhibition of binding by the 25 bp fragments was observed for fragment 4 (data not shown) addition of fragment 6.1 inhibited GlnR-binding to fragment 6 (Figure 5C and D). Thus, for *amtB*, two binding sites were experimentally verified, one located 75–100 bp upstream (fragment 6.1) and the other 100–150 bp upstream of the gene’s start codon (fragment 4).

The binding sequences show similarity to the proposed binding motif [11]; however, for a more detailed characterization of GlnR binding sites in *M. smegmatis*, further analyses are necessary. Besides competitive gel retardation assays, especially ChIP and ChIP-chip analyses as presented recently for *S. venezuelae*[15] might be carried out.

### Growth of M. smegmatis on different nitrogen sources

Deduced from the increased transcript levels of several genes in the wild-type compared to *ΔglnR* strain MH1 (Table 1), a number of new substances were proposed as nitrogen sources for *M. smegmatis.* For ammonium, alanine, asparagine, glutamic acid, glutamine, nitrate, nitrite and urea a function as nitrogen source was already shown [16-18]; however, due to the experimental set-up, the experiments carried out here allowed conclusions about the GlnR-dependent or -independent regulation of assimilation pathways.

**Figure 6:**
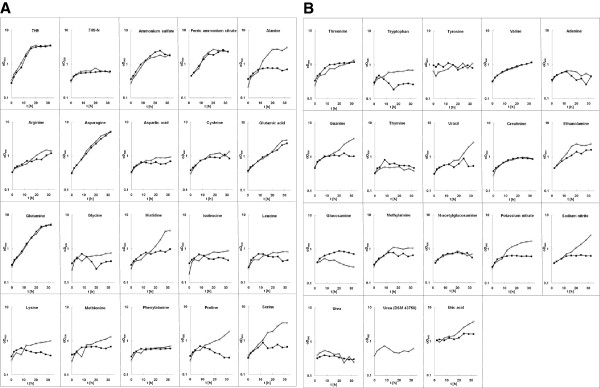
**Growth of *M. smegmatis *in various nitrogen sources.** Wild-type SMR5 and *glnR* deletion strain MH1 were grown in the presence of the indicated substances (10 mM final concentration) as sole nitrogen source. Standard 7H9 medium was used as positive control; 7H9 lacking any nitrogen source as negative control (7H9-N). **(A)** Putative nitrogen sources deduced from microarray results, **(B)** putative nitrogen sources used by closely related species.

Growth experiments were carried out using the various substances tested as sole nitrogen source and comparing wild-type and *ΔglnR* strain MH1 (Figure 6). 7H9 medium was used as positive control and nitrogen-free 7H9 (7H9-N) as negative control. Standard 7H9 medium contains ammonium sulphate, ferric ammonium citrate and glutamic acid as nitrogen sources. When ammonium sulphate and ferric ammonium citrate were tested alone, a reduced growth rate and final OD_600_ was observed, independently from the presence or absence of GlnR, reflecting that ammonium (NH_4_^+^) is membrane-permeable in its unprotonated form ammonia (NH_3_) and that under the growth conditions tested, ammonium was not limiting.

Further growth tests revealed that metabolism of alanine, histidine, proline and serine was significantly impaired in strain MH1 compared to the wild-type. Consequently, uptake and/or metabolism of these amino acids seem to be directly or indirectly regulated by GlnR. No significant influence of a *glnR* deletion was observed when arginine, asparagine, glutamate, glutamine and valine were used as sole nitrogen source, indicating a GlnR-independent regulation of the metabolism of these amino acids. Aspartate, cysteine, glycine isoleucine, leucine, lysine, methionine, phenylalanine, threonine, tryptophan and tyrosine are poor or not suitable nitrogen sources for *M. smegmatis* under the conditions tested here (Figure 6A).

The increased levels of various genes connected to purine and pyrimidine uptake and metabolism (Table 1) in response to nitrogen starvation prompted us to test these also as nitrogen sources. Guanine and uracil supported growth in a GlnR-dependent manner, which is in accordance with increased mRNA levels for (*msmeg_5730* 45 x, *msmeg_1293* 15 x, *msmeg_6660* 11 x, encoding putative uracil transport proteins; Table 1), while adenine and thymine were no suitable nitrogen sources.

Additionally other substrates, which are for example used by corynebacteria or streptomycetes, were tested (Figure 6B). GlnR-dependent growth was observed when ethanolamine, nitrate, nitrite or uric acid were added, which is in accordance with the transcriptome data. Growth was not supported by creatinine, glucosamine, methylamine, *N*-acetylglucosamine and urea. The latter finding was especially puzzling, since *M. smegmatis* is characterized as urease-positive and gene clusters for two ureases and one urea transporter were described based on bioinformatic analyses [12]. Furthermore, when transcript patterns of nitrogen-supplied and -starved wild-type cells were compared in this study, increased mRNA levels were observed at least for one gene of a putative *urt* (urea transporter) gene cluster (*msmeg_2982* 136 x) and for a putative urea ammonium lyase (*msmeg_2187* 3 x). To exclude strain-specific variations, type-strain DSM 43796 (equivalent to ATCC 19420 and NCTC 8159) was tested under the same growth conditions as SMR5 and MH1. Also in this case, supply of urea alone did not support growth in 7H9-N independent of the addition for example of different trace element solutions (data not shown). Subsequently carried out enzyme activity measurements revealed a urease activity of 1 μmol of ammonium released min^-1^ (mg protein)^-1^, independent of growth of strains SMR5 and DSM 43756 in nitrogen surplus or incubated for 30 minutes under nitrogen starvation. This correlates with data obtained for *C. glutamicum*, which shows a urease activity of 0.9 μmol min^-1^ (mg protein)^-1^ in complex medium and a urease activity of 0.9-2.2 μmol min^-1^ (mg protein)^-1^ when ammonium is used as single nitrogen source. In this bacterium, however, urease activity is increased to 7.8 μmol min^-1^ (mg protein)^-1^, when cells are exposed to nitrogen starvation [19,20]. Despite the fact that urease activity is not upregulated in response to starvation, *M. smegmatis* shows at least low urease activity and the reason for the complete growth deficiency with urea as single nitrogen source remains unclear.

In summary, as a soil bacterium and opportunistic human pathogen, *M. smegmatis* is able to metabolize a wide variety of nitrogen sources. Transcriptome and growth data presented here hint to a major role of GlnR in ammonium, amino acid, nitrate/nitrite and purine/pyrimidine assimilation, while additionally other regulators seem to be present as well as indicated by the GlnR-independent metabolism of several amino acids as nitrogen sources.

### Analysis of the AmtR regulon

**Figure 7:**
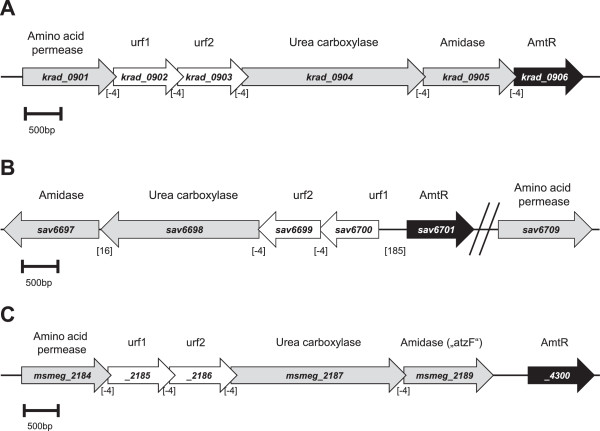
**Co-occurrence of *amtR *and AmtR-regulated genes in selected *Actinobacteria*.** Genomic map of the highly conserved AmtR operon in *K. radiotolerans***(A)**, *S. avermitilis***(B)** and *M. smegmatis***(C)**. The arrows indicate length and transcriptional orientation of annotated genes which are depicted by the respective orf number. Numbers in square brackets show the lengths of intergenic regions in bp. While the putative *K. radiotolerans* operon shows a subsequent series of genes with overlapping reading frames, the *amtR* gene is transcribed in opposite direction in *S. avermitilis* and the amino acid permease-encoding gene is located elsewhere, while in *M. smegmatis*, the *amtR* gene is not co-localized with the putatively AmtR-regulated genes (see Table 3 for details).

The experiments described above supported the idea that GlnR is a major nitrogen regulator in *M. smegmatis*, which consequently leads to the question of AmtR function in this organism. In a first approach to characterize the AmtR regulon, a bioinformatic analysis was carried out to identify AmtR-controlled genes in *M. smegmatis.* For this purpose, a co-occurrence analysis was carried out using the genome information of ten actinobacterial genomes, i.e. *Arthrobacter aurescens*, *C. michiganensis*, *C. efficiens*, *Gordonia bronchialis*, *K. radiotolerans*, *M. smegmatis*, *Nocardia farcinica*, *Rhodococcus jostii*, *Streptomyces avermitilis* and *Tsukumurella paurometabola.* From these genomes, one conserved operon was extracted, which (i) shows a high degree of co-occurrence with the *amtR* gene, (ii) shows co-localization with *amtR* in *A. aurescens*, *Rhodococcus* sp. RHA1 and *S. avermitilis* and (iii) seems to be involved in nitrogen metabolism based on their annotation (Table 3). *K. radiotolerans* features the most condensed AmtR gene cluster (Figure 7); all open reading frames of this putative operon feature overlapping start and stop codons (GTGA or ATGA). While AmtR and the amino acid permease are highly conserved and well consistently annotated in the different genomes, the exact functions of unknown reading frames urf1 and urf2, the urea carboxylase and the amidase are highly speculative and deduced from annotations (automatic and manual), domain analyses and PFAM searches. The location of these unknown reading frames in direct genomic neighborhood to the putative urea carboxylase-encoding gene is highly conserved in all investigated genomes, while the locations of the genes coding for amino acid permease and the amidase are more variable. Particularly the *amtR* gene can be found in close vicinity as well as completely elsewhere in the respective genome sequences (Table 3, Figure 7).

**Table 3:** Distribution of AmtR and screening for AmtR-regulated genes

**Organism (Reference)**	**Putative function and gene identifier**			
	**Amino acid permease**	**Urea carboxylase**	**Amidase**	**AmtR**
*Arthrobacter aurescens* TC1 [21]	*AAur_0190*	*AAur_0187*	*AAur_0186*	*AAur0192*
*Corynebacterium efficiens* YS-314 [22]	*ce0711*	*ce0713*	*ce0710*	*ce0939*
*Clavibacter michi-ganensis* NCPPB 382 [23]	*CMM_0123*	*CMM_0120*	*CMM_2824*	*CMM_0119*
*Gordonia bronchialis* DSM 43247 (NCBI GenBank, acc. no. CP001802.1)	*Gbro_3891*	*Gbro_3888*	*Gbro_3887*	*Gbro_0881*
*Kineococcus radiotolerans* SRS30216 [24]	*Krad_0901*	*Krad_0904*	*Krad_0905*	*Krad_0906*
*Mycobacterium smegmatis* mc^2^ 155 (NCBI GenBank, acc. no. CP000480)	*msmeg_2184*	*msmeg_2187*	*msmeg_2189*	*msmeg_4300*
*Nocardia farcinica* IFM10152 [25]	*nfa22220*	*nfa22190*	*nfa22180*	*nfa22230*
*Rhodococcus jostii* RHA1 (NCBI GenBank, acc. no. CP000431.1)	*RHA1_ro06919*	*RHA1_ro06922*	*RHA1_ro02136*	*RHA1_ro06918*
*Streptomyces avermitilis* MA-4680 [26]	*sav6709*	*sav6698*	*sav6697*	*sav6701*
*Tsukamurella paurometabola* DSM20162 (NCBI GenBank, acc. no. CP001966)	*Tpau_1591*	*Tpau_1594*	*Tpau_1595*	*Tpau_1590*

The genome sequences of various *Actinobacteria* were screened for an *amtR* gene and co-occurrence of putatively AmtR-regulated genes (hypothetical urea carboxylase-associated unknown reading frames not shown, for details see text).

At least four of the identified six putatively AmtR-regulated *M. smegmatis* genes showed increased transcript levels in response to starvation in the wild-type and seem to be under control of GlnR (*msmeg_2184* 48 x, *msmeg_2185* 13 x, *msmeg_2186* 32 x, *msmg_2187* 3 x, *msmg_2189* 4 x; Table 2). To investigate transcription control of these genes, a strain, designated YL1, with in frame deletion of gene fragment (*amtR*_*DBD*_) encoding the DNA binding domain of AmtR and an *amtR*_*DBD*_/*glnR* double deletion strain, designated YL2, were constructed, respectively. These strains were analyzed in respect to nitrogen-starvation-dependent transcription.

**Figure 8:**
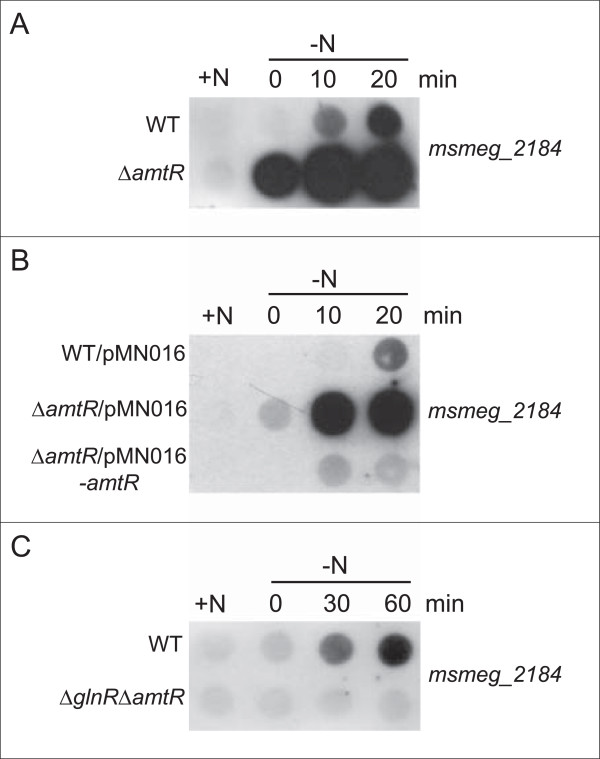
**Influence of AmtR on target gene expression.** Hybridization experiments were carried out with RNA isolated from nitrogen-supplied cells and after different intervals of nitrogen starvation and a probe specific for *msmeg_2184* mRNA. **(A)** Comparison of wild-type SMR5 (WT) and *amtR* deletion strain YL1 (∆*amtR*). **(B)** Complementation of *amtR* deletion strain YL1 with plasmid pMN016-*amtR* (∆*amtR*/pMN016-*amtR*); wild-type and strain YL1 transformed with vector pMN016 (WT/pMN016 and ∆*amtR*/pMN016) were used as control. **(C)** Influence of a *glnR/amtR* double deletion (∆*glnR*∆*amtR*).

RNA hybridization experiments with RNA isolated from the wild-type and *amtR* deletion strain YL1, isolated from nitrogen-supplied cells and after different intervals of nitrogen starvation showed significantly stronger signals for the proposed AmtR-regulated genes *msmeg_2184* (Figure 8A), *msmeg_2187* and *msmeg_2189* (data not shown) in the mutant; however, nitrogen control was still intact. Wild-type regulation for all genes was restored by a plasmid carrying *amtR*, while the vector alone did not complement the deletion (see Figure 8B for *msmeg_2184*, others: data not shown). Nitrogen-dependent regulation of AmtR-regulated genes was abolished in *glnR* deletion strain MH1 (see Figure 8C *msmeg_2184*, others: data not shown), indicating that AmtR is a secondary regulator of the master regulator GlnR. This model is in accordance with the transcriptome analyses presented above (Table 1).

**Figure 9:**
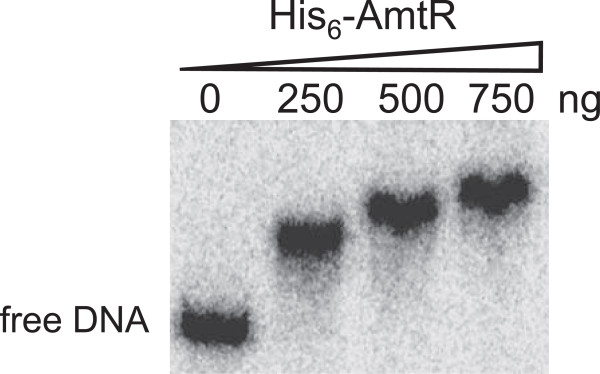
**Test of AmtR binding by gel retardation assays.** The indicated amounts of purified AmtR proteins were added to a ^32^P-labeled *msmeg_2184* promoter fragment (437 bp) in the presence of 2ug sperm DNA (non-specific).

To verify direct AmtR-DNA interaction, *M. smegmatis* AmtR was overexpressed in *Eschericha coli*, purified and applied in gel retardation experiments. Binding of AmtR was tested for upstream DNA of *msmeg_2184* and binding to the *msmeg_2184* upstream region was demonstrated (Figure 9). Since TetR-type regulators often bind small effector molecules that induce release of the repressor from its binding site, addition of putative AmtR effectors deduced from the putative function of target genes, i.e. urea and different amino acids, were tested in various concentrations (0.5 to 5 mM) but had no influence on binding to the *msmeg_2184* upstream region (data not shown).

For a more global analysis of the AmtR regulon, DNA microarray experiments were carried out. RNA samples isolated from the wild-type and *amtR* deletion strain YL1 grown under nitrogen limitation were hybridized, and also RNA samples from *amtR* deletion mutant YL1 grown with and without nitrogen source were analyzed. No further AmtR targets were observed (data not shown), indicating that AmtR controls only a very small regulon, which is part of the GlnR modulon in *M. smegmatis*.

**Figure 10:**
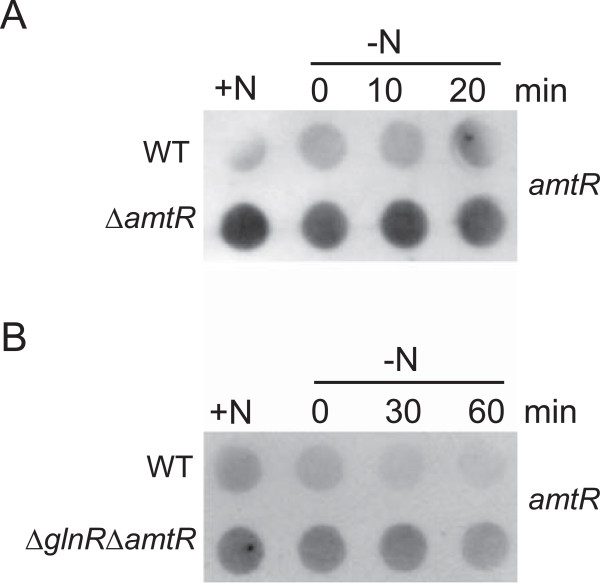
**Autoregulation of AmtR.** Hybridization experiments were carried out with RNA isolated from nitrogen-supplied cells and after different intervals of nitrogen starvation and a probe specific for *amtR* mRNA. Since the deletion in *amtR* only comprises the DNA binding domain encoding nucleotides, hybridization of the *amtR* probe is possible with RNA isolated from strains YL1 (∆*amtR*) and YL2 (∆*glnR*∆*amtR*).

In an additional approach, autoregulation of AmtR was analyzed. This regulatory mechanism typical for TetR-type regulators [27] was in fact observed (Figure 10). Since the *amtR* deletion only comprised the part of the gene encoding the DNA binding domain, an *amtR* target was detectable, which was stronger in the deletion mutant YL1 compared to the wild-type (Figure 10A). Induction of nitrogen starvation or an additional deletion of *glnR* (Figure 10B) had no effect, indication a nitrogen-independent regulation of AmtR expression.

## Conclusions

Deprivation of nitrogen leads to growth arrest in *M. smegmatis* and induces not only specific transcriptional alterations in respect to genes encoding nitrogen-related proteins, but a general starvation response at the transcriptional level. This includes changes in the mRNA levels of several hundred genes encoding transporters, proteins involved in nitrogen metabolism and regulation, energy generation and protein turnover etc. and was for example also observed in other *Actinobacteria* such as *C. glutamicum*[14,28].

Based on the DNA microarray data obtained for *M. smegmatis* wild-type SMR5 and *glnR* mutant MH1 and growth experiments, GlnR seems to play a crucial role in nitrogen metabolism in *M. smegmatis*., including the uptake and assimilation of ammonium, different amino acids (alanine, histidine, proline, serine), nitrate, nitrite, uric acid, ethanolamine, guanine and uracil, while, AmtR, the master regulator of nitrogen control in corynebacteria plays a minor role. All in all, nitrogen control in this species resembles carbon catabolite repression (CCR) in enterobacteria (for recent reviews on CCR in bacteria, see [29,30]. Like CcpA in CCR, GlnR seems to be the global regulator and functions as an activator in response to a starvation stimulus, while AmtR is a specific repressor of a small regulon, corresponding to LacI in CCR.

In addition to the GlnR-dependent regulation of nitrogen metabolism, genes not associated with nitrogen were indicated as putative GlnR targets by the DNA microarray analyses carried out. While these genes were not in the focus of this study, it can be assumed that the regulatory function of GlnR might not be restricted to nitrogen metabolism in *M. smegmatis*. This has also been suggested in similar studies focusing on *S. coelicolor* GlnR [3], where genes involved in carbon metabolism, stress response and antibiotic biosynthesis were also found to be GlnR-controlled confirming the regulatory function of GlnR beyond nitrogen metabolism. Future work will concentrate on the question, whether the observed transcription changes of non-nitrogen-related genes are directly GlnR-regulated or the result of perturbations caused by the *glnR* deletion, which indirectly affect expression.

## Methods

### Bioinformatics analyses

For bioinformatics analysis of the AmtR regulon, a screening and co-occurrence approach was carried out. Genome sequences were obtained from the NCBI Genbank (ftp://ftp.ncbi.nih.gov/genbank/genomes/Bacteria/) and manually investigated with the software program Artemis (Sanger Institute). Homology analyses were performed with the microbial genomes BLAST databases at the NCBI and JCVI CMR servers. Advanced protein sequence predictions were done at the EMBL SMART domain analysis server and co-occurrence screenings were performed with the EMBL STRING interaction networks database.

### Growth conditions

Bacteria were routinely grown at 37°C in baffled flasks under agitation. Mycobacterial strains were grown in Middlebrook 7H9 liquid medium (Difco Laboratories; per 900 ml approx. 0.5 g ammonium sulfate, 0.5 g L-glutamic acid, 0.1 g sodium citrate, 1.0 mg pyridoxine, 0.5 mg biotin, 2.5 g disodium phosphate, 1.0 g mono potassium phosphate, 0.04 g ferric ammonium citrate, 0.05 g magnesium sulfate, 0.5 mg calcium chloride, 1.0 mg zinc sulfate, 1.0 mg copper sulfate), supplemented with 0.2% glycerol and 0.05% Tween 80 or on Middlebrook 7H9 medium (Difco Laboratories) with 1.5% agar supplemented with 0.2% glycerol and 0.05% Tween 80. If appropriate, antibiotics were added in the following concentrations: hygromycin (200 μg ml^−1^ for *E. coli*; 50 μg ml^−1^ for *M. smegmatis*), kanamycin (30 μg ml^−1^ for *E. coli*; 10 μg ml^−1^ for *M. smegmatis*) and streptomycin (400 μg ml^−1^ for *M. smegmatis*). In order to study the effects of nitrogen starvation, a fresh *M. smegmatis* culture was used to inoculate 7H9 medium for overnight growth. This culture, with an overnight OD_600_ of approximately three to four, was used to inoculate fresh 7H9 medium to an OD_600_ of approximately 0.2, and cells were grown for 10 to 11 hours until the exponential growth phase was reached (OD_600_ approximately 0.6 to 0.8). To induce nitrogen starvation, cells were harvested by centrifugation, washed and resuspended in pre-warmed 7H9 medium without nitrogen source (7H9 lacking ammonium sulfate and glutamic acid and containing iron citrate instead of iron ammonium citrate). For control, the unstarved cells were harvested, washed, and transferred using 7H9 medium.

### General molecular biology techniques

For plasmid isolation, transformation, and cloning standard techniques were used [31]. *E. coli* strain DH5αMCR [32] was used as cloning host. Plasmids were subsequently brought into competent *M. smegmatis* cells by electroporation. Chromosomal DNA was extracted from 100 ml cultures grown to stationary phase as described [33]. DNA sequence analyses were carried out using Big Dye® Terminator V3.1 Cycle Sequencing kit (Perkin Elmer).

### Construction of an *amtR* deletion strain

To inactivate the AmtR function, a mutant with in-frame deletion of the DNA fragment (*amtR*_*DBD*_) encoding AmtR DNA-binding domain (DBD) (amino acid residue 26 to 72) was generated as described earlier [11]. Briefly, two DNA fragments (1 kb up- and downstream of *amtR*_*DBD*_) were amplified *via* PCR using chromosomal DNA from *M. smegmatis* strain SMR5 as a template. *Swa*I and *Pac*I restriction sites were introduced into the primer sequences used for amplification of the upstream arm and *Spe*I and *Pme*I sites into the primer sequences used for amplification of the downstream arm (Additional file 1: Table S3)*.* The PCR products were then cloned in vector pML814 (ColE1 origin, *FRT*-*hyg*-*FRT*, *rpsL*, Amp^R^, Hyg^R^, 6220 bp, general deletion vector), resulting in the recombinant plasmid pML814Δ*amtR*_*DBD*,_ which carried a *FRT*-*hyg*-*FRT* expression cassette [34] flanked by the upstream and downstream arms. *M. smegmatis* SMR5 was transformed with pML814Δ*amtR*_*DBD*_ and the transformants were first selected on hygromycin-containing plates to obtain single crossovers [34]. After verification of the single crossover event *via* colony PCR, cells were further selected on hygromycin/streptomycin containing plates. Clones on these plates should have lost the vector and have the integrated *FRT-hyg-FRT* cassette in the chromosome. After verification of the double crossover event *via* PCR, the FLP recombinase was used to specifically remove the *hyg* gene from the chromosome, thus generating a marker-free deletion mutant. Selection of clones was performed using hygromycin/streptomycin-containing plates. Deletion of *amtR*_*DBD*_ in the resulting strain MH2 was verified by PCR and Southern blotting (data not shown).

On basis of the mutant strain YL1, *glnR* was further deleted as described earlier [11], generating the mutant strain YL2, with the inactivation of both *amtR*_*DBD*_ and *glnR*, which was confirmed by PCR and Southern blotting (data not shown).

### Construction of *amtR* carrying plasmids

For complementation assays, the *amtR* gene was amplified by PCR using chromosomal DNA of *M. smegmatis* as template and the primers amtRcom-fw/amtRcom-rev (see Additional file 1: Table S3). The PCR products were ligated to plasmid pMN016 (p_smyc_-*mspA*, ColE1 origin, pAL5000 origin, Hyg^R^, 6164 bp, [35]) between the *Swa*I and *Pac*I sites. For the overexpression of AmtR protein in *E. coli*, the *amtR* gene was amplified by PCR (for primers, see Additional file 1: Table S3) and ligated to plasmid pQE70 (Qiagen, Hilden) to add a C-terminal His_6_-tag to the expressed protein, leading to plasmid pQE-*amtR-His*_*6*_. The resulting plasmids pMN016-*amtR* and pQE-*amtR-His*_*6*_ were sequenced for verification (data not shown).

### Construction of antisense probes

For the generation of antisense probes, internal DNA fragments of the corresponding genes were amplified by PCR (primers for the different probes are listed in supplementary materials, Additional file 1: Table S3). The reverse primers encoded the promoter region for T7 polymerase, which allowed *in vitro* transcription of probes using T7 polymerase.

### RNA preparation, hybridization analyses

*M. smegmatis* RNA was prepared from 6 ml culture samples using the NucleoSpin RNA II Kit (Macherey Nagel, Düren). When necessary, a second DNase digestion was performed with Turbo-DNase (Ambion) to completely remove the chromosomal DNA. RNA samples were finally stored at −80°C.

Antisense probes with a size between 0.2 and 0.5 kb for the analysis of gene transcription were generated by PCR and subsequent labelling with DIG RNA-labelling mix (Roche, Mannheim) and T7 polymerase (NEB, Frankfurt). RNA (1 μg per time point) was spotted onto nylon membranes using a Schleicher & Schuell (Dassel) Minifold I Dot Blotter. Hybridization of digoxigenin-labelled RNA probes was detected with X-ray films (Amersham Hyperfilm MP; GE Healthcare) using alkaline phosphatase-conjugated anti-digoxigenin Fab fragments and CSPD as light-emitting substrate as recommended by the supplier (Roche, Mannheim). All experiments were carried out at least twice with independent cultures (biological replicates).

### Real-time reverse transcriptase PCR

For real-time RT-PCR, a MyiQ Single-Color Real-Time PCR Detection System (BioRad, Munich), the iScript One-Step RT-PCR Kit with SYBR Green (Biorad, Munich), 1 μM primers, and 100 ng of template RNA were used. Reverse transcription was carried out at 50°C for 10 min, the reverse transcriptase was inactivated and the polymerase activated by 5 min incubation at 95°C, PCR was carried out by 45 cycles of the following program: DNA denaturation for 10 sec at 95°C, primer annealing for 15 sec at 57°C, and DNA polymerization for 10 sec at 72°C. The PCR reaction was followed by a melting curve program (81 x 55–100°C with a heating rate of 1°C per 10 sec) and then a cooling program at 25°C. No-template controls were run with all reactions. Data were analyzed using the MyiQ Single-Color Real-Time PCR Detection System software.

### Design of DNA microarrays, RNA quality control, labelling, hybridization and transcriptome analysis

Whole genome expression analyses were performed using custom *M. smegmatis* strain mc^2^ 155 8x15K microarrays (Agilent, Santa Clara). Based on annotated genes downloaded from the NCBI genome browser (http://www.ncbi.nlm.nih.gov/genomes), two specific probes per gene were designed using the eArray software (https://earray.chem.agilent.com).

Total RNA from two biological replicates of each bacterial strain or condition was isolated as described above and purified using RNeasy Mini Spin Columns (Qiagen, Hilden, Germany). RNA quantity was measured with the ND-100 Spectrophotometer v3.3.0 (NanoDrop Technologies). RNA integrity was verified using an Agilent RNA 6000 Nano Chip on an Agilent 2100 BioAnalyzer as recommended by manufacturer’s protocol (Agilent RNA 6000 Nano Assay Protocol2). Reverse transcription of 300 ng total RNA was carried out, using a random T7N9 primer [36]. Further sample labeling and hybridization was essentially performed as described in the two-color microarray-based gene expression analysis protocol provided by Agilent including the two-color RNA spike-in kit (v5.7, 2008; Agilent Technologies, Santa Clara). For each replicate the cy3/cy5 dye combination was flipped between control and experimental sample. Slides were scanned on the Agilent Microarray Scanner with extended dynamic range (XDR) at high resolution (5 μm). Data sets were extracted by the feature extraction software package (v9.5.3.1/ Agilent Technologies) using a standard protocol. Data were analyzed using the GeneSpring XI software (Agilent Technologies) with standard settings. Statistically significantly deregulated genes (> 2-fold change in comparison to respective control and p-value < 0.05) were identified using the *t-test against zero* function of GeneSpring XI including the Benjamini-Hochberg multiple test correction. The microarray datasets were deposited in GEO database (http://www.ncbi.nlm.nih.gov/geo/) under record number GSE30236.

### Purification of GlnR and gel retardation experiments

For gel shift assays, protein extracts from *E. coli* Rosetta2 carrying plasmid pMal-c2-*glnR* were prepared. Cells were cultivated in LB medium containing antibiotics as described, harvested by centrifugation (4,000 x g, 15 min, 4°C) and suspended in 20 mM Tris, 200 mM NaCl, 1 mM EDTA, pH (HCl) 7.4 (2 ml g^-1^ cell weight) containing lysozyme (2 mg ml^-1^) and Complete protease inhibitor as recommended by the supplier (Roche, Mannheim). Cells were subsequently disrupted by ultrasonic treatment. Cell debris was removed by centrifugation (14,000 x g, 30 min, 4°C) and the protein extract was loaded on a 10 ml amylose resin affinity column (GE Healthcare, Munich) in a chromatography device (Äkta prime, GE Healthcare, Munich). Washing and subsequent elution with 20 mM Tris, 200 mM NaCl, 1 mM EDTA, 20 mM maltose, pH (HCl) 7.4 were carried out as recommended by the supplier of the amylose resin matrix (GE Healthcare, Munich); the purified protein was stored at 4°C.

Target DNA for the gel shift assays was synthesized by PCR (primers listed in Additional file 1: Table S1) and purified by agarose gel electrophoresis. For labelling of the DNA and the setup of the reaction mixture for the gel shift assay, the DIG Gel Shift Kit (Roche, Mannheim) was used following the supplier’s protocol. Separation by gel electrophoresis was performed in native 6% polyacrylamide gels (Anamed Electrophorese GmbH, Darmstadt) using 0.5 x TBE buffer as running buffer. Subsequently, the labelled DNA was blotted on a nylon membrane (Roche, Mannheim) by electro-blotting as described in the protocol of the DIG Gel Shift Kit (Roche, Mannheim). For detection of the labelled DNA, X-ray films were used.

### Purification of AmtR and electrophoretic mobility shift assays

For purification of AmtR protein, *E. coli* BL21 (DE3) carrying plasmid pQE-*amtR*-His_6_ was used to inoculate 200 ml LB and the cultures were induced with 0.5% α-lactose at an OD_600_ of approximately 0.6 and grown for 12 h at 16°C. The cells were harvested by centrifugation, washed twice with lysis buffer (20 mM Tris–HCl, 500 mM KCl, 10% glycerol, pH 7.9), and resuspended in binding buffer (20 mM Tris–HCl, 500 mM KCl, 10% glycerol, 10 mM imidazole, pH 7.9). The cell suspension was stored on ice for 30 min and then lysed by sonication. The lysate were centrifuged at 15,000 x g for 60 min at 4°C and applied to a 1-mL Ni Sepharose column (GE Healthcare) following 1 h incubation on ice. After washing with 20 ml of binding buffer, the column was washed with 20 ml washing buffer A (20 mM Tris–HCl, 500 mM KCl, 10% glycerol, 20 mM imidazole, pH 7.9) and B (20 mM Tris–HCl, 500 mM KCl, 10% glycerol, 50 mM imidazole, pH7.9), respectively. The bound protein was eluted with elution buffer (20 mM Tris–HCl, 500 mM KCl, 10% glycerol, 500 mM imidazole, pH 7.9) and the purity of His-tagged protein was checked by 12% sodium dodecyl sulfate polyacrylamide gel electrophoresis (SDS-PAGE). The purified protein was stored at −80°C.

Electrophoretic mobility shift assays (EMSAs) were performed according to the method described before with some modifications [37]. The promoter region of *msmeg_2184* (437 bp) was amplified using *M. smegmatis* genomic DNA as template and the primer pair, msmeg_2184pfw/msmeg_2184prev (see Additional file 1: Table S3). PCR product was labeled at the 5’ ends with [γ-^32^P]ATP using T4 polynucleotide kinase (Promega, China), and purified using illustra ProbeQuant G-50 Micro Columns (GE Healthcare, UK). For EMSA, the ^32^P-labelled DNA probe (1,000 cpm) was incubated individually with various amounts of His_6_-AmtR protein in 20 μl binding assays, containing 2 μg sperm DNA, 20 mM Tris–HCl (pH 7.9), 25 mM KCl, 1 mM dithiothreitol (DTT), 5 mM MgCl_2_, 0.5 mg ml^-1^ calf BSA and 5% glycerol. After incubation at 25°C for 30 min, protein-bound and -free probe DNA were separated by electrophoresis on non-denaturing 6% polyacrylamide gels at 10 V cm^-1^ with a running buffer 0.5 x TBE (40 mM Tris–HCl, pH 8.0, 20 mM boric acid and 1 mM EDTA). After electrophoresis, gels were dried and scanned with a FLA-7000 phosphoimager (FujiFilm Corporation, Japan).

### Availability of supporting data

The datasets supporting the results of this article are available in the GEO repository (http://www.ncbi.nlm.nih.gov/geo/) under record number GSE30236.

## Competing interests

The authors declare that they have no competing interests.

## Authors’ contributions

NJ carried out RNA experiments and growth tests; YL was responsible for AmtR experimental work, while bioinformatic analyses of AmtR were carried out by JA. SR supported the DNA microarray experiments, FT, SS and AB were responsible for general experimental design and supervision of experiments. The manuscript was mainly written by NJ and AB. All authors read and approved the final manuscript.

## Supplementary Material

Additional file 1: Table S1. List of all 231 genes with decreased transcript levels in the *M. smegmatis* wild type strain SMR5 under nitrogen starvation. **Table S2.** List of all 284 genes with increased transcript levels in the *M. smegmatis* wild type strain SMR5 under nitrogen starvation. **Table S3.** Primers used in this study. Restriction sites are highlighted bold. Click here for file
