# Supplementary material for: Nitrogen starvation-induced transcriptome alterations and influence of transcription regulator mutants in Mycobacterium smegmatis
Source: BMC Res Notes. 2013 Nov 22;6:482. doi: 10.1186/1756-0500-6-482 (PMC4222082; doi:10.1186/1756-0500-6-482)
Supplement: Additional file 1: Table S1. — List of all 231 genes with decreased transcript levels in the M. smegmatis wild type strain SMR5 under nitrogen starvation. Table S2. List of all 284 genes with increased transcript levels in the M. smegmatis wild type strain SMR5 under nitrogen starvation. Table S3. Primers used in this study. Restriction sites are highlighted bold. [file 1756-0500-6-482-S1.docx]

**Supplementary data**

**Table S1: List of all 231 genes with decreased transcript levels in the *M. smegmatis* wild type strain SMR5 under nitrogen starvation.**

| Gene identifier | Fold change | Annotation |
| --- | --- | --- |
| *msmeg_2536* | 3.01 | 3-oxoacyl-[acyl-carrier-protein] reductase |
| *msmeg_3509* | 3.01 | conserved hypothetical protein |
| *msmeg_0016* | 3.01 | conserved domain protein |
| *msmeg_3792* | 3.01 | ribosomal protein L35 (*rpmI*) |
| *msmeg_0985* | 3.02 | sugar transporter family protein |
| *msmeg_6238* | 3.02 | putative two-component system sensor kinase |
| *msmeg_0531* | 3.03 | putative acyl-CoA dehydrogenase |
| *msmeg_3363* | 3.03 | regulatory protein, TetR |
| *msmeg_6313* | 3.04 | queuine tRNA-ribosyltransferase (*tgt*) |
| *msmeg_0401* | 3.04 | putative non-ribosomal peptide synthase |
| *msmeg_3364* | 3.05 | RhtB family transporter |
| *msmeg_2349* | 3.05 | glycosyl hydrolase, family 57 |
| *msmeg_5061* | 3.06 | bacterial extracellular solute-binding protein |
| *msmeg_3710* | 3.08 | cytochrome b561 family protein |
| *msmeg_4627* | 3.08 | nucleoside diphosphate kinase |
| *msmeg_0788* | 3.10 | putative conserved membrane protein |
| *msmeg_6034* | 3.11 | hypothetical protein |
| *msmeg_2963* | 3.12 | bacterial extracellular solute-binding proteins, family 5 |
| *msmeg_2853* | 3.12 | conserved hypothetical protein |
| *msmeg_0313* | 3.13 | phosphogluconate dehydratase (*edd*) |
| *msmeg_6315* | 3.13 | lipoprotein LpqH |
| *msmeg_5525* | 3.13 | succinyl-CoA synthetase, beta subunit (*sucC*) |
| *msmeg_1019* | 3.14 | ribonucleoside-diphosphate reductase, alpha subunit |
| *msmeg_5060* | 3.15 | ABC transporter, permease protein SugA |
| *msmeg_2499* | 3.15 | ABC transporter, membrane spanning protein |
| *msmeg_4644* | 3.16 | molybdopterin-guanine dinucleotide biosynthesis protein A |
| *msmeg_3539* | 3.17 | hypothetical protein |
| *msmeg_2042* | 3.18 | phosphotransferase enzyme family protein |
| *msmeg_1401* | 3.19 | translation elongation factor Tu (*tuf*) |
| *msmeg_1870* | 3.19 | conserved hypothetical protein |
| *msmeg_5897* | 3.21 | virulence factor mce family protein |
| *msmeg_6458* | 3.23 | glutamate synthase, small subunit |
| *msmeg_1524* | 3.24 | DNA-directed RNA polymerase, alpha subunit (*rpoA*) |
| *msmeg_1525* | 3.24 | 50S ribosomal protein L17 |
| *msmeg_4958* | 3.25 | diaminopimelate decarboxylase (*lysA*) |
| *msmeg_4646* | 3.25 | pyruvate synthase |
| *msmeg_6404* | 3.25 | UDP-galactopyranose mutase (*glf*) |
| *msmeg_3159* | 3.26 | methylmalonyl-CoA mutase large subunit |
| *msmeg_4656* | 3.27 | sugar ABC transporter ATP-binding protein |
| *msmeg_1341* | 3.27 | MaoC family protein |
| *msmeg_0082* | 3.27 | conserved hypothetical protein |
| *msmeg_6402* | 3.28 | PAP2 superfamily protein |
| *msmeg_4111* | 3.29 | alpha-methylacyl-CoA racemase |
| *msmeg_1963* | 3.30 | putative transcriptional regulatory protein |
| *msmeg_4118* | 3.31 | acyl-CoA dehydrogenase |
| *msmeg_0140* | 3.32 | probable conserved mce associated membrane protein |
| *msmeg_5091* | 3.32 | hypothetical protein |
| *msmeg_0840* | 3.32 | hypothetical protein |
| *msmeg_1339* | 3.33 | ribosomal protein L33 (*rpmG*) |
| *msmeg_4114* | 3.35 | naphthoate synthase (*menB*) |
| *msmeg_5432* | 3.35 | peptidyl-tRNA hydrolase (*pth*) |
| *msmeg_2533* | 3.36 | hypothetical protein |
| *msmeg_2348* | 3.36 | glycosyl transferase, group 1 family protein |
| *msmeg_1023* | 3.36 | integral membrane transporter |
| *msmeg_1256* | 3.37 | hypothetical protein |
| *msmeg_4087* | 3.37 | major facilitator superfamily |
| *msmeg_3962* | 3.38 | lactate 2-monooxygenase |
| *msmeg_5222* | 3.39 | GTP-binding protein YchF (*ychF*) |
| *msmeg_6050* | 3.40 | solute-binding lipoprotein |
| *msmeg_5968* | 3.41 | polysaccharide biosynthesis protein |
| *msmeg_6947* | 3.41 | chromosomal replication initiator protein DnaA (*dnaA*) |
| *msmeg_0059* | 3.41 | ATPase, AAA family |
| *msmeg_3051* | 3.43 | guanylate kinase |
| *msmeg_6236* | 3.43 | two-component system, regulatory protein |
| *msmeg_6021* | 3.43 | xylose isomerase (*xylA*) |
| *msmeg_1398* | 3.43 | ribosomal protein S12 (*rpsL*) |
| *msmeg_0061* | 3.44 | ftsk-spoiiie family protein |
| *msmeg_1255* | 3.47 | UvrD-Rep helicase |
| *msmeg_5211* | 3.48 | aminotransferase class-III |
| *msmeg_6542* | 3.48 | B12 binding domain protein |
| *msmeg_0078* | 3.48 | hypothetical protein |
| *msmeg_6903* | 3.49 | transcriptional regulator, PadR family protein |
| *msmeg_5901* | 3.49 | TrnB2 protein |
| *msmeg_0787* | 3.51 | bacterial extracellular solute-binding proteins, family 3 |
| *msmeg_5199* | 3.51 | putative acyl-CoA dehydrogenase |
| *msmeg_2820* | 3.51 | hypothetical protein |
| *msmeg_0055* | 3.52 | hypothetical protein |
| *msmeg_5198* | 3.52 | carnitinyl-CoA dehydratase |
| *msmeg_3708* | 3.53 | catalase |
| *msmeg_0238* | 3.53 | O-acetylhomoserine-O-acetylserine sulfhydrylase |
| *msmeg_6758* | 3.56 | transport integral membrane protein |
| *msmeg_5923* | 3.56 | acetyl-CoA acetyltransferase |
| *msmeg_4645* | 3.56 | alpha oxoglutarate ferredoxin oxidoreductase, beta subunit |
| *msmeg_4086* | 3.57 | nitrilotriacetate monooxygenase component A (*ssuD*) |
| *msmeg_6400* | 3.57 | probable conserved transmembrane protein |
| *msmeg_5896* | 3.58 | virulence factor Mce family protein |
| *msmeg_5899* | 3.58 | virulence factor Mce family protein |
| *msmeg_0410* | 3.60 | MmpL protein |
| *msmeg_6638* | 3.61 | methyltransferase (*metE*) |
| *msmeg_5895* | 3.63 | virulence factor mce family protein |
| *msmeg_3047* | 3.64 | carbamoyl-phosphate synthase, large subunit (*carB*) |
| *msmeg_3161* | 3.64 | lipoprotein, putative |
| *msmeg_1473* | 3.64 | ribosomal protein L30 (*rpmD*) |
| *msmeg_5057* | 3.65 | conserved hypothetical protein |
| *msmeg_0622* | 3.65 | putative DNA-binding protein |
| *msmeg_0108* | 3.67 | acyl-CoA dehydrogenase |
| *msmeg_6759* | 3.68 | glycerol kinase (*glpK*) |
| *msmeg_0062* | 3.68 | ftsk-spoiiie family protein |
| *msmeg_3599* | 3.69 | sugar-binding transcriptional regulator, LacI family |
| *msmeg_4116* | 3.71 | 3-hydroxybutyryl-CoA dehydrogenase |
| *msmeg_4561* | 3.72 | ABC Fe3^+^-siderophores transporter, periplasmic binding protein |
| *msmeg_3197* | 3.73 | lipase |
| *msmeg_1805* | 3.73 | conserved hypothetical protein |
| *msmeg_6904* | 3.74 | myo-inositol-1-phosphate synthase |
| *msmeg_1474* | 3.75 | ribosomal protein L15 (*rplO*) |
| *msmeg_5371* | 3.78 | ectoine-hydroxyectoine ABC transporter, ATP-binding protein |
| *msmeg_6307* | 3.79 | glutamine-binding periplasmic protein |
| *msmeg_3598* | 3.80 | periplasmic sugar-binding proteins |
| *msmeg_6942* | 3.80 | membrane protein OxaA |
| *msmeg_0132* | 3.82 | conserved hypothetical protein |
| *msmeg_0620* | 3.82 | pe family protein |
| *msmeg_4756* | 3.83 | holo-(acyl-carrier-protein) synthase (*acpS*) |
| *msmeg_1704* | 3.83 | ABC transporter |
| *msmeg_0138* | 3.84 | virulence factor Mce family protein |
| *msmeg_2130* | 3.84 | putative acyl-CoA dehydrogenase |
| *msmeg_1705* | 3.85 | D-xylose transport ATP-binding protein XylG |
| *msmeg_2073* | 3.86 | CAIB-BAIF family protein |
| *msmeg_6761* | 3.87 | glycerol-3-phosphate dehydrogenase 2 |
| *msmeg_3713* | 3.87 | hypothetical protein |
| *msmeg_0380* | 3.88 | MmpS4 protein |
| *msmeg_6585* | 3.89 | acyl-CoA dehydrogenase |
| *msmeg_4115* | 3.90 | 3-hydroxybutyryl-CoA dehydrogenase |
| *msmeg_1349* | 3.92 | dgpf domain family |
| *msmeg_4560* | 3.92 | periplasmic binding protein |
| *msmeg_0239* | 3.92 | O-acetylhomoserine-O-acetylserine sulfhydrylase |
| *msmeg_1342* | 3.92 | conserved hypothetical protein |
| *msmeg_0786* | 3.99 | serine-threonine protein kinase |
| *msmeg_0068* | 4.00 | probable conserved transmembrane protein |
| *msmeg_0080* | 4.00 | conserved hypothetical protein |
| *msmeg_1340* | 4.00 | conserved hypothetical protein |
| *msmeg_3094* | 4.02 | oxidoreductase, inc-binding dehydrogenase family |
| *msmeg_4083* | 4.04 | putative monooxygenase |
| *msmeg_4530* | 4.05 | sulfate ABC transporter, ATP-binding protein (*cysA*) |
| *msmeg_4531* | 4.05 | sulfate ABC transporter, permease protein CysW (*cysW*) |
| *msmeg_1448* | 4.06 | integral membrane transporter |
| *msmeg_4557* | 4.09 | ABC transporter, ATP-binding protein |
| *msmeg_3602* | 4.10 | ribose transport ATP-binding protein RbsA |
| *msmeg_3092* | 4.11 | transcriptional regulator, sugar-binding family |
| *msmeg_3603* | 4.11 | oxidoreductase, inc-binding dehydrogenase family |
| *msmeg_5902* | 4.11 | domain of unknown function superfamily |
| *msmeg_1466* | 4.12 | ribosomal protein L24 (*rplX*) |
| *msmeg_2503* | 4.14 | lipoprotein, putative |
| *msmeg_3090* | 4.18 | ribose transport system permease protein RbsC |
| *msmeg_6018* | 4.21 | xylose transport system permease protein XylH |
| *msmeg_1467* | 4.23 | 50S ribosomal protein L5 |
| *msmeg_0520* | 4.23 | porin |
| *msmeg_0075* | 4.24 | conserved hypothetical protein |
| *msmeg_1470* | 4.29 | 50S ribosomal protein L6 |
| *msmeg_5483* | 4.31 | porin |
| *msmeg_6229* | 4.31 | glycerol kinase (*glpK*) |
| *msmeg_6057* | 4.32 | MspD protein |
| *msmeg_0143* | 4.34 | probable conserved mce associated membrane protein |
| *msmeg_1469* | 4.34 | ribosomal protein S8 (*rpsH*) |
| *msmeg_0550* | 4.35 | sulfonate binding protein |
| *msmeg_4301* | 4.37 | acyl-CoA synthase |
| *msmeg_4532* | 4.38 | sulfate ABC transporter, permease protein CysT (*cysT*) |
| *msmeg_0382* | 4.39 | putative transport protein |
| *msmeg_6391* | 4.41 | propionyl-CoA carboxylase beta chain |
| *msmeg_6450* | 4.41 | hypothetical protein |
| *msmeg_4559* | 4.42 | ABC transporter, membrane spanning protein |
| *msmeg_0381* | 4.42 | Mmp14a protein |
| *msmeg_0079* | 4.45 | hypothetical protein |
| *msmeg_1807* | 4.46 | acetyl--propionyl-coenyme A carboxylase alpha chain |
| *msmeg_1443* | 4.47 | ribosomal protein L16 (*rplP*) |
| *msmeg_0142* | 4.49 | conserved hypothetical protein |
| *msmeg_6649* | 4.50 | conserved hypothetical protein |
| *msmeg_6459* | 4.53 | ferredoxin-dependent glutamate synthase 1 |
| *msmeg_0131* | 4.54 | AMP-binding enzyme, putative |
| *msmeg_2534* | 4.56 | putative carboxylesterase protein |
| *msmeg_5058* | 4.59 | ABC transporter, ATP-binding protein SugC |
| *msmeg_2619* | 4.65 | efflux protein |
| *msmeg_1741* | 4.65 | TetR-family transcriptional regulator |
| *msmeg_3058* | 4.70 | lipoprotein, nlpa family |
| *msmeg_1472* | 4.71 | ribosomal protein S5 (*rpsE*) |
| *msmeg_1442* | 4.72 | ribosomal protein S3 (*rpsC*) |
| *msmeg_1436* | 4.74 | ribosomal protein L3 (*rplC*) |
| *msmeg_3538* | 4.74 | cyclopropane-fatty-acyl-phospholipid synthase 1 |
| *msmeg_1435* | 4.76 | ribosomal protein S10 (*rpsJ*) |
| *msmeg_3095* | 4.77 | D-ribose-binding periplasmic protein |
| *msmeg_0141* | 4.80 | probable conserved mce associated transmembrane protein |
| *msmeg_0965* | 4.81 | porin |
| *msmeg_2121* | 4.84 | multiphosphoryl transfer protein (MTP) |
| *msmeg_1999* | 4.85 | hypothetical protein |
| *msmeg_1444* | 4.85 | ribosomal protein L29 (*rpmC*) |
| *msmeg_4210* | 4.86 | secreted protein |
| *msmeg_0549* | 4.87 | ABC transporter, permease protein |
| *msmeg_5816* | 4.88 | conserved hypothetical protein |
| *msmeg_1471* | 4.88 | ribosomal protein L18 (*rplR*) |
| *msmeg_1350* | 4.91 | cyclopropane-fatty-acyl-phospholipid synthase 1 |
| *msmeg_0134* | 4.97 | virulence factor Mce family protein |
| *msmeg_1465* | 5.03 | ribosomal protein L14 (*rplN*) |
| *msmeg_6760* | 5.11 | conserved hypothetical protein |
| *msmeg_0084* | 5.12 | phosphocarrier protein hpr |
| *msmeg_1437* | 5.12 | ribosomal protein L4-L1 family (*rplD*) |
| *msmeg_1445* | 5.15 | 30S ribosomal protein S17 |
| *msmeg_0135* | 5.18 | virulence factor Mce family protein |
| *msmeg_5059* | 5.21 | ABC transporter, permease protein SugB |
| *msmeg_1439* | 5.21 | ribosomal protein L2 (*rplB*) |
| *msmeg_1468* | 5.28 | ribosomal protein S14p-S29e (*rpsN*) |
| *msmeg_1438* | 5.39 | ribosomal protein L23 (*rplW*) |
| *msmeg_1441* | 5.44 | 50S ribosomal protein L22 |
| *msmeg_1440* | 5.64 | ribosomal protein S19 (*rpsS*) |
| *msmeg_5418* | 5.95 | iron permease FTR1 |
| *msmeg_0133* | 6.02 | ABC-transporter integral membrane protein |
| *msmeg_6762* | 6.03 | transcriptional regulator |
| *msmeg_5591* | 6.06 | conserved hypothetical protein |
| *msmeg_0137* | 6.09 | virulence factor mce family protein |
| *msmeg_5412* | 6.12 | immunogenic protein MPT63 |
| *msmeg_6392* | 6.23 | polyketide synthase |
| *msmeg_0085* | 6.39 | PTS system, Fru family, IIABC components |
| *msmeg_1583* | 6.58 | chaperonin GroL (*groL*) |
| *msmeg_6242* | 6.65 | alcohol dehydrogenase, iron-containing |
| *msmeg_2535* | 6.74 | dehydrogenase-reductase SDR family member 10 |
| *msmeg_0880* | 6.92 | chaperonin GroL (*groL*) |
| *msmeg_0020* | 7.19 | periplasmic binding protein |
| *msmeg_4326* | 7.50 | acyl carrier protein (*acpP*) |
| *msmeg_5435* | 7.53 | acyl-CoA synthase |
| *msmeg_4329* | 7.55 | propionyl-CoA carboxylase beta chain |
| *msmeg_5420* | 7.77 | Tat-translocated enzyme |
| *msmeg_1582* | 7.97 | chaperonin GroS (*groS*) |
| *msmeg_4325* | 7.98 | malonyl CoA-acyl carrier protein transacylase |
| *msmeg_1812* | 8.04 | conserved hypothetical protein |
| *msmeg_4328* | 8.25 | 3-oxoacyl-[acyl-carrier-protein] synthase 2 |
| *msmeg_0530* | 8.35 | short chain dehydrogenase |
| *msmeg_4757* | 9.08 | fatty acid synthase |
| *msmeg_1813* | 9.18 | propionyl-CoA carboxylase beta chain |
| *msmeg_4327* | 9.32 | 3-oxoacyl-[acyl-carrier-protein] synthase 1 |
| *msmeg_1810* | 9.98 | hypothetical protein |
| *msmeg_1811* | 11.24 | septum formation protein Maf (*maf*) |
| *msmeg_5589* | 12.60 | manganese transport protein MntH |
| *msmeg_5419* | 14.63 | lipoprotein |

**Table S2: List of all 284 genes with increased transcript levels in the *M. smegmatis* wild type strain SMR5 under nitrogen starvation.**

| Gene identifier | Fold change | Annotation |
| --- | --- | --- |
| *msmeg_6612* | 3.01 | ATPase, MoxR family (*moxR*) |
| *msmeg_5180* | 3.01 | conserved hypothetical protein |
| *msmeg_2416* | 3.01 | conserved hypothetical protein |
| *msmeg_0451* | 3.02 | oxidoreductase, FAD-linked |
| *msmeg_6506* | 3.02 | nicotinamidase-pyrainamidase |
| *msmeg_0541* | 3.02 | hypothetical protein |
| *msmeg_3561* | 3.04 | glutamine synthetase, catalytic domain (*glnA3*) |
| *msmeg_3137* | 3.05 | oxidoreductase |
| *msmeg_2113* | 3.05 | hypothetical protein |
| *msmeg_1251* | 3.05 | conserved hypothetical protein |
| *msmeg_2916* | 3.06 | DNA-binding response regulator, PhoP family |
| *msmeg_1312* | 3.06 | hypothetical protein |
| *msmeg_5842* | 3.06 | conserved hypothetical protein |
| *msmeg_1079* | 3.07 | hypothetical protein |
| *msmeg_0755* | 3.07 | cobalt-inc-cadmium resistance protein |
| *msmeg_3254* | 3.07 | RDD family, putative |
| *msmeg_2112* | 3.07 | secreted protein |
| *msmeg_2376* | 3.08 | conserved hypothetical protein |
| *msmeg_2589* | 3.11 | conserved hypothetical protein |
| *msmeg_1301* | 3.12 | NanT3 |
| *msmeg_5014* | 3.12 | copper-translocating P-type ATPase |
| *msmeg_1883* | 3.12 | glycine betaine transporter OpuD |
| *msmeg_3816* | 3.13 | excinuclease ABC, B subunit (*uvrB*) |
| *msmeg_0171* | 3.13 | histone deacetylase superfamily |
| *msmeg_0450* | 3.14 | hypothetical protein |
| *msmeg_5308* | 3.18 | conserved hypothetical protein |
| *msmeg_0267* | 3.20 | esterase |
| *msmeg_2428* | 3.21 | DNA-binding protein |
| *msmeg_4567* | 3.21 | conserved hypothetical protein |
| *msmeg_0752* | 3.22 | fructose-bisphosphate aldolase, class II (*fbaA*) |
| *msmeg_1552* | 3.22 | ethanolamine permease (*eat*) |
| *msmeg_4546* | 3.23 | oxidoreductase |
| *msmeg_4417* | 3.25 | methionine-S-sulfoxide reductase (*msrA*) |
| *msmeg_3912* | 3.26 | acetoacetyl-CoA reductase |
| *msmeg_5355* | 3.27 | hypothetical protein |
| *msmeg_3372* | 3.28 | transcriptional regulator, ArsR family |
| *msmeg_2960* | 3.28 | preprotein translocase, YajC subunit (*yajC*) |
| *msmeg_5935* | 3.29 | ATP-dependent DNA helicase |
| *msmeg_1755* | 3.29 | anti-sigma factor, ChrR |
| *msmeg_4569* | 3.29 | conserved hypothetical protein |
| *msmeg_3138* | 3.29 | thioredoxin (*trx*) |
| *msmeg_0393* | 3.30 | Fmt protein |
| *msmeg_1273* | 3.31 | conserved hypothetical protein |
| *msmeg_2696* | 3.31 | putative conserved membrane alanine rich protein |
| *msmeg_0223* | 3.32 | conserved hypothetical protein |
| *msmeg_6212* | 3.33 | hemerythrin HHE cation binding domain subfamily, putative |
| *msmeg_5917* | 3.36 | conserved hypothetical protein |
| *msmeg_5401* | 3.36 | conserved hypothetical protein |
| *msmeg_6477* | 3.38 | methionine-S-sulfoxide reductase (*msrA*) |
| *msmeg_0172* | 3.38 | probable conserved transmembrane protein, putative |
| *msmeg_4171* | 3.38 | ribose transport system permease protein RbsC |
| *msmeg_0850* | 3.38 | conserved hypothetical protein |
| *msmeg_2115* | 3.39 | conserved hypothetical protein |
| *msmeg_5647* | 3.41 | conserved hypothetical protein |
| *msmeg_3417* | 3.41 | conserved hypothetical protein |
| *msmeg_1501* | 3.42 | methyltransferase, putative, family |
| *msmeg_6616* | 3.44 | S-(hydroxymethyl)glutathione dehydrogenase |
| *msmeg_0222* | 3.45 | conserved hypothetical protein |
| *msmeg_1802* | 3.46 | ChaB protein |
| *msmeg_6659* | 3.47 | hypothetical protein |
| *msmeg_2187* | 3.47 | urea amidolyase |
| *msmeg_6416* | 3.51 | phosphoglycerate mutase family protein |
| *msmeg_0051* | 3.52 | transcription factor WhiB family |
| *msmeg_3289* | 3.57 | gp61 protein |
| *msmeg_1097* | 3.58 | glycosyl transferase, group 2 family protein |
| *msmeg_6879* | 3.59 | ABC-type Nat permease for neutral amino acids NatD |
| *msmeg_4465* | 3.59 | cutinase |
| *msmeg_6876* | 3.60 | branched chain amino acid transport ATP-binding protein |
| *msmeg_2913* | 3.65 | hydrolase |
| *msmeg_1595* | 3.65 | putative oxidoreductase |
| *msmeg_1769* | 3.68 | UsfY protein |
| *msmeg_3661* | 3.69 | conserved hypothetical protein |
| *msmeg_6878* | 3.70 | inner-membrane translocator |
| *msmeg_6727* | 3.72 | amino acid permease-associated region |
| *msmeg_4456* | 3.74 | conserved hypothetical protein |
| *msmeg_4294* | 3.75 | glutamine synthetase, type I (*glnA2*) |
| *msmeg_6332* | 3.75 | amino acid ABC transporter, permease protein |
| *msmeg_6880* | 3.76 | hydrophobic amino acid ABC transporter, putative |
| *msmeg_0637* | 3.78 | iron-sulfur binding oxidoreductase |
| *msmeg_0585* | 3.79 | L-carnitine dehydratase-bile acid-inducible protein F |
| *msmeg_1762* | 3.79 | piperideine-6-carboxylic acid dehydrogenase |
| *msmeg_3022* | 3.80 | transglycosylase associated protein |
| *msmeg_1090* | 3.82 | amidase |
| *msmeg_2925* | 3.84 | permease membrane component |
| *msmeg_1151* | 3.85 | DNA-binding protein |
| *msmeg_1605* | 3.85 | phosphate transport system regulatory protein PhoU (*phoU*) |
| *msmeg_0074* | 3.88 | IS1549, transposase |
| *msmeg_1089* | 3.89 | hypothetical protein |
| *msmeg_3862* | 3.91 | FxsA cytoplasmic membrane protein |
| *msmeg_6213* | 3.91 | Manganese containing catalase |
| *msmeg_6877* | 3.94 | branched-chain amino acid transporter, ATP-binding protein |
| *msmeg_5484* | 3.96 | conserved hypothetical protein |
| *msmeg_1698* | 3.96 | putative ammonia monooxygenase superfamily |
| *msmeg_0911* | 3.97 | isocitrate lyase (*aceA*) |
| *msmeg_3564* | 3.99 | bacterioferritin (*bfr*) |
| *msmeg_3419* | 3.99 | hypothetical protein |
| *msmeg_6254* | 4.01 | hypothetical protein |
| *msmeg_2659* | 4.01 | alanine dehydrogenase (*ald*) |
| *msmeg_4382* | 4.02 | dehydrogenase-reductase SDR family member 10 |
| *msmeg_5078* | 4.04 | glucose-1-phosphate adenylyltransferase (*glgC*) |
| *msmeg_4570* | 4.04 | conserved hypothetical protein |
| *msmeg_1292* | 4.04 | FAD binding domain in molybdopterin dehydrogenase protein |
| *msmeg_5334* | 4.05 | conserved hypothetical protein |
| *msmeg_0072* | 4.06 | IS1549, transposase, interruption-C |
| *msmeg_1991* | 4.10 | isovaleryl-CoA dehydrogenase |
| *msmeg_1088* | 4.13 | glutamyl-tRNA(Gln)-aspartyl-tRNA (Asn) amidotransferase |
| *msmeg_1411* | 4.15 | universal stress protein family |
| *msmeg_3902* | 4.16 | ATPase, AAA family |
| *msmeg_2756* | 4.16 | conserved hypothetical protein |
| *msmeg_2343* | 4.16 | methylesterase |
| *msmeg_6355* | 4.17 | hypothetical protein |
| *msmeg_1787* | 4.18 | RsbW protein |
| *msmeg_5542* | 4.22 | transcriptional regulator, HTH_3 family |
| *msmeg_6480* | 4.23 | putative transcriptional regulatory protein |
| *msmeg_5682* | 4.24 | conserved hypothetical protein |
| *msmeg_1768* | 4.27 | conserved hypothetical protein |
| *msmeg_3439* | 4.27 | hypothetical protein |
| *msmeg_5646* | 4.28 | conserved hypothetical protein |
| *msmeg_5016* | 4.29 | conserved domain protein |
| *msmeg_5764* | 4.30 | putative cyanamide hydratase |
| *msmeg_6354* | 4.33 | serine esterase, cutinase family |
| *msmeg_0231* | 4.33 | conserved hypothetical protein |
| *msmeg_2755* | 4.38 | conserved hypothetical protein |
| *msmeg_4073* | 4.39 | DNA-binding protein |
| *msmeg_6733* | 4.42 | hydrolase, carbon-nitrogen family |
| *msmeg_3471* | 4.44 | GTP cyclohydrolase |
| *msmeg_1773* | 4.49 | conserved hypothetical protein |
| *msmeg_1412* | 4.50 | amino acid permease |
| *msmeg_1530* | 4.51 | integral membrane protein |
| *msmeg_5333* | 4.56 | hypothetical protein |
| *msmeg_6874* | 4.63 | aldehyde dehydrogenase |
| *msmeg_5374* | 4.66 | glutamate--ammonia ligase |
| *msmeg_4499* | 4.67 | hypothetical protein |
| *msmeg_3722* | 4.67 | bifunctional coenyme PQQ synthesis protein C-D |
| *msmeg_1296* | 4.70 | uricase |
| *msmeg_5327* | 4.72 | hypothetical protein |
| *msmeg_5648* | 4.73 | hypothetical protein |
| *msmeg_4765* | 4.80 | transcriptional regulator, MerR family |
| *msmeg_0230* | 4.80 | conserved hypothetical protein |
| *msmeg_1771* | 4.82 | methylase, putative |
| *msmeg_6467* | 4.85 | starvation-induced DNA protecting protein |
| *msmeg_1791* | 4.90 | UsfY protein |
| *msmeg_5331* | 4.91 | UDP-glucoronosyl and UDP-glucosyl transferase family |
| *msmeg_5402* | 4.92 | dehydrogenase DhgA |
| *msmeg_4542* | 4.93 | oligopeptide transport integral membrane protein |
| *msmeg_3255* | 5.00 | DoxX subfamily, putative |
| *msmeg_1950* | 5.01 | conserved hypothetical protein |
| *msmeg_1767* | 5.08 | conserved hypothetical protein |
| *msmeg_1508* | 5.15 | amino acid permease-associated region |
| *msmeg_1032* | 5.18 | hypothetical protein |
| *msmeg_5617* | 5.24 | immunogenic protein MPT63 |
| *msmeg_5558* | 5.27 | hypothetical protein |
| *msmeg_0429* | 5.31 | putative ferric uptake regulator |
| *msmeg_5375* | 5.32 | GntR-family transcriptional regulator |
| *msmeg_1085* | 5.33 | dipeptide transport system permease protein DppB |
| *msmeg_1134* | 5.38 | putative protease HtpX |
| *msmeg_0566* | 5.44 | aliphatic amidase |
| *msmeg_6264* | 5.44 | putative oxidoreductase |
| *msmeg_2569* | 5.48 | oxidoreductase, 2OG-Fe(II) oxygenase family |
| *msmeg_2695* | 5.52 | 35 kDa protein |
| *msmeg_1295* | 5.54 | transthyretin |
| *msmeg_5341* | 5.56 | dipeptidyl aminopeptidase-acylaminoacyl peptidase |
| *msmeg_1789* | 5.58 | conserved hypothetical protein |
| *msmeg_1786* | 5.67 | stas domain, putative |
| *msmeg_1774* | 5.71 | conserved hypothetical protein |
| *msmeg_2523* | 5.81 | efflux ABC transporter, permease protein, putative |
| *msmeg_4989* | 5.94 | sensor histidine kinase |
| *msmeg_2798* | 5.95 | hypothetical protein |
| *msmeg_6223* | 5.97 | TetR family transcriptional repressor LfrR |
| *msmeg_6478* | 6.02 | putative cytochrome P450 135B1 |
| *msmeg_1788* | 6.09 | conserved hypothetical protein |
| *msmeg_1772* | 6.12 | conserved hypothetical protein |
| *msmeg_5275* | 6.15 | permease of the major facilitator superfamily |
| *msmeg_4298* | 6.16 | 3-methyl-2-oxobutanoate hydroxymethyltransferase (*panB*) |
| *msmeg_6579* | 6.19 | conserved hypothetical protein |
| *msmeg_5071* | 6.25 | conserved hypothetical protein |
| *msmeg_5729* | 6.25 | hydantoin racemase |
| *msmeg_1234* | 6.26 | taurine import ATP-binding protein TauB |
| *msmeg_1794* | 6.38 | dehydrogenase |
| *msmeg_5072* | 6.40 | extracytoplasmic function alternative sigma factor |
| *msmeg_1052* | 6.44 | amino acid carrier protein |
| *msmeg_3371* | 6.45 | short-chain dehydrogenase-reductase SDR |
| *msmeg_0436* | 6.68 | allophanate hydrolase subunit 1 |
| *msmeg_1766* | 6.76 | conserved hypothetical protein |
| *msmeg_4290* | 6.85 | glutamine synthetase, type I (*glnA*) |
| *msmeg_6507* | 6.89 | glycogen debranching enzyme GlgX (*glgX*) |
| *msmeg_1131* | 6.94 | tryptophan-rich sensory protein |
| *msmeg_4381* | 6.94 | amidase |
| *msmeg_2751* | 7.05 | hypothetical protein |
| *msmeg_1792* | 7.06 | conserved hypothetical protein |
| *msmeg_3403* | 7.10 | formamidase |
| *msmeg_1076* | 7.12 | lipoprotein, putative |
| *msmeg_1990* | 7.48 | conserved hypothetical protein |
| *msmeg_6881* | 7.49 | transcriptional regulator, GntR family |
| *msmeg_5485* | 7.50 | molybdopterin biosynthesis protein |
| *msmeg_5015* | 7.51 | secreted protein |
| *msmeg_6263* | 7.60 | glutamate synthase family protein |
| *msmeg_0434* | 7.70 | aminoglycoside 2-N-acetyltransferase (AAC(2)-Id) |
| *msmeg_0778* | 7.81 | putative transcriptional regulator |
| *msmeg_6225* | 7.89 | proton antiporter efflux pump |
| *msmeg_2748* | 7.96 | soluble pyridine nucleotide transhydrogenase (*sthA*) |
| *msmeg_0780* | 7.99 | phosphotransferase enzyme family protein |
| *msmeg_6116* | 8.21 | conserved hypothetical protein |
| *msmeg_3402* | 8.33 | cytosine permease, putative |
| *msmeg_0435* | 8.36 | allophanate hydrolase subunit 2 |
| *msmeg_6660* | 8.37 | permease, cytosine-purines, uracil, thiamine, allantoin family |
| *msmeg_2694* | 8.40 | transcriptional regulator, XRE family |
| *msmeg_2752* | 8.63 | sigma factor SigB |
| *msmeg_1951* | 8.68 | conserved domain protein |
| *msmeg_1031* | 8.71 | conserved hypothetical protein |
| *msmeg_2157* | 8.77 | hypothetical protein |
| *msmeg_5356* | 9.03 | hypothetical protein |
| *msmeg_1030* | 9.06 | monooxygenase |
| *msmeg_1596* | 9.09 | transcriptional regulator |
| *msmeg_2525* | 9.58 | amino acid permease superfamily |
| *msmeg_6115* | 9.65 | phosphoglycerate dehydrogenase |
| *msmeg_5083* | 9.80 | conserved hypothetical protein |
| *msmeg_6817* | 9.97 | RNA polymerase sigma factor, sigma-70 family |
| *msmeg_6262* | 10.48 | FwdC-FmdC family protein |
| *msmeg_1790* | 10.58 | conserved hypothetical protein |
| *msmeg_2159* | 10.65 | conserved hypothetical protein |
| *msmeg_4990* | 10.73 | DNA-binding response regulator |
| *msmeg_2978* | 10.88 | ABC transporter ATP-binding protein |
| *msmeg_1086* | 11.03 | ABC transporter permease protein |
| *msmeg_2981* | 11.22 | branched-chain amino acid ABC-type transport system |
| *msmeg_1415* | 11.30 | AsnC-family transcriptional regulator |
| *msmeg_1770* | 11.53 | conserved hypothetical protein |
| *msmeg_1293* | 11.94 | xanthine-uracil permeases family protein |
| *msmeg_1764* | 11.97 | L-lysine-epsilon aminotransferase |
| *msmeg_2185* | 12.49 | conserved hypothetical protein |
| *msmeg_3400* | 13.26 | glutamyl-tRNA (Gln) amidotransferase subunit A |
| *msmeg_1185* | 14.14 | transcriptional regulator, AsnC family |
| *msmeg_2980* | 14.39 | putative membrane protein |
| *msmeg_0586* | 14.47 | stas domain, putative |
| *msmeg_5486* | 14.84 | peptidase S1 and S6, chymotrypsin-Hap |
| *msmeg_4206* | 15.10 | Molybdopterin oxidoreductase |
| *msmeg_3401* | 15.18 | LamB-YcsF family protein |
| *msmeg_6259* | 15.21 | ammonium transporter (*amt1*) |
| *msmeg_1101* | 15.80 | hypothetical protein |
| *msmeg_6261* | 16.46 | glutamine amidotransferase, class II |
| *msmeg_2979* | 16.59 | ABC transporter ATP-binding protein |
| *msmeg_1597* | 16.64 | Transcription factor WhiB |
| *msmeg_1087* | 16.86 | oligopeptide ABC transporter ATP-binding protein |
| *msmeg_2427* | 16.95 | protein PII uridylyltransferase (*glnD*) |
| *msmeg_0572* | 18.21 | conserved hypothetical protein |
| *msmeg_4637* | 19.71 | conserved hypothetical protein |
| *msmeg_4635* | 19.87 | ammonium transporter family protein (*amtA*) |
| *msmeg_5344* | 21.36 | hypothetical protein |
| *msmeg_1413* | 21.40 | ornithine--oxo-acid transaminase (*rocD*) |
| *msmeg_5358* | 21.65 | acetamidase-formamidase family |
| *msmeg_0781* | 21.68 | amino acid permease |
| *msmeg_5342* | 21.72 | conserved hypothetical protein |
| *msmeg_4501* | 21.94 | sodium:dicarboxylate symporter |
| *msmeg_6735* | 21.95 | amino acid permease, putative |
| *msmeg_5359* | 23.33 | cyanate hydratase (*cynS*) |
| *msmeg_1184* | 24.13 | serine esterase, cutinase family |
| *msmeg_4636* | 24.53 | hypothetical protein |
| *msmeg_5343* | 24.71 | conserved hypothetical protein |
| *msmeg_5084* | 24.96 | glycosyl transferase, group 2 family protein |
| *msmeg_2524* | 24.97 | ABC transporter, ATP-binding protein |
| *msmeg_2522* | 25.50 | efflux ABC transporter, permease protein |
| *msmeg_0569* | 27.14 | flavoprotein involved in K+ transport |
| *msmeg_4638* | 27.62 | vanillate O-demethylase oxidoreductase |
| *msmeg_2186* | 27.93 | conserved hypothetical protein |
| *msmeg_5765* | 28.31 | globin |
| *msmeg_2425* | 29.43 | ammonium transporter (*amtB*) |
| *msmeg_6816* | 30.09 | molybdopterin oxidoreductase |
| *msmeg_6260* | 30.44 | glutamine synthetase, type III (*glnT*) |
| *msmeg_0432* | 31.62 | uroporphyrinogen-III synthetase |
| *msmeg_2426* | 31.84 | nitrogen regulatory protein PII (*glnK*) |
| *msmeg_5730* | 33.27 | permease for cytosine-purines, uracil, thiamine, allantoin |
| *msmeg_0779* | 33.83 | short-chain dehydrogenase-reductase SDR |
| *msmeg_2982* | 35.17 | putative periplasmic binding protein |
| *msmeg_1082* | 37.64 | putative response regulator |
| *msmeg_0428* | 37.81 | nitrite reductase [NAD(P)H] small subunit (*nirD*) |
| *msmeg_1987* | 41.10 | conserved hypothetical protein |
| *msmeg_6734* | 44.58 | dibenothiophene desulfuriation enyme A |
| *msmeg_1084* | 48.16 | peptide-opine-nickel uptake family ABC transporter |
| *msmeg_0427* | 49.99 | nitrite reductase [NAD(P)H], large subunit (*nirB*) |
| *msmeg_5329* | 51.18 | conserved hypothetical protein |
| *msmeg_0571* | 54.10 | hydrolase, carbon-nitrogen family |
| *msmeg_0433* | 60.20 | nitrite extrusion protein (*narK3*) |
| *msmeg_2526* | 66.11 | copper methylamine oxidase |
| *msmeg_1414* | 98.67 | amidinotransferase |

**Table S3: Primers used in this study.** Restriction sites are highlighted bold.

| Name | Sequence |
| --- | --- |
| Real time RT PCR: |  |
| RT-3084-fw | 5‘-GTCGTCGAGTCCACCGGCA-3‘ |
| RT-3084-rev | 5‘-CGTCGGTGGCCGGCG-3‘ |
| RT-0428nirD-fw | 5‘-GTGCCTACGACTTCCTCATACCGG-3‘ |
| RT-0428nirD-rev | 5‘-GCGTGCACCGAACCGTCATC-3‘ |
| RT-0779-fw | 5‘-CGGTGGCGGGCCGTTCGAC-3‘ |
| RT-0779-rev | 5‘-TCGCTGACATCGGTCTGCACGCC-3‘ |
| RT-1293-fw | 5‘-GGTGACAAATCCGCACACCGGCG-3‘ |
| RT-1293-rev | 5‘-GATCCGCTGGATCGCGACGATG-3‘ |
| RT-2184-fw | 5‘-GGCTCGGCCGGTTCGAGTCG-3‘ |
| RT-2184-rev | 5‘-GGCCCGCCGAAGCCGAAACC-3‘ |
| RT-2425amtB-fw | 5‘-CGGTGATCACCGTCGCGCTGA-3‘ |
| RT-2425amtB-rev | 5‘-AAATACACCAGGGTGACCCACAGGC-3‘ |
| RT-2426glnK-fw | 5‘-GATCGTCAAGCCGTTCACGCTGG-3‘ |
| RT-2426glnK-rev | 5‘-CTGGCGCCCGTAGCCCTGAA-3‘ |
| RT-2427glnD-fw | 5‘-GCGACCTGCCGCCGCGAG-3‘ |
| RT-2427glnD-rev | 5‘-CGCGACACCCGGGTGGTG-3‘ |
| RT-2522-fw | 5‘-CTCGGTGCTCGGGATCGCG-3‘ |
| RT-2522-rev | 5‘-GACGTCGGTCACCGAGTCGGC-3‘ |
| Sonde-2526-fw | 5‘-CGCCGACAACCGGCCCGC-3‘ |
| RT-2526-rev | 5‘-GTTCCACCCGTCGACGGTGG-3‘ |
| RT-2982urtA-fw | 5‘-CTCCGAGGTCACCGTCCG-3‘ |
| RT-2982urtA-rev | 5‘-GTCCTCGCCGATCAGTTCG-3‘ |
| RT-3400-fw | 5‘-CGATCAGCTCTTTCCACCGACTCGT-3‘ |
| RT-3400-rev | 5‘-TCGATGGAGTCGAGTGTTGCGATGC-3‘ |
| Sonde-4290glnA-fw | 5‘-CTTCGAGTCCGACGGCAGCG-3‘ |
| RT-4290glnA-rev | 5‘-GTCGCGCAGGTCGACGTAGTG-3‘ |
| RT-4294glnA2-fw | 5‘-CGTCGCCCGGCCTGATCCG-3‘ |
| RT-4294glnA2-rev | 5‘-GGGCATGGTGATGTCGCAGAACATG-3‘ |
| RT-4635amtA-fw | 5‘-GGTTCCTCGCCTACGAGGTCG-3‘ |
| RT-4635amtA-rev | 5‘-TAGAAGGACGCGATGATGGTGGCC-3‘ |
| RT-5730-fw | 5‘-CTGATCGGCATCTCGATCGTCAACG-3‘ |
| RT-5730-rev | 5‘-CGAACACGGTGCGGCAGATCAC-3‘ |
| RT-6259amt1-fw | 5‘-TGTTCGGTACGTCCTACGGCGGAT-3‘ |
| RT-6259amt1-rev | 5‘-ACCGGCAGCCCGTCGACGGTG-3‘ |
| RT-6261-fw | 5‘-GACCTGCTCGAACGTGTCAAGGC-3‘ |
| RT-6261-rev | 5‘-GCGCACGCGGATGGCCCACA-3‘ |
| RT-6816-fw | 5‘-AGATCATCGCGCAGGGTCTCGTC-3‘ |
| RT-6816-rev | 5‘-CGTTCGGGTGGGTAGTTCGCG-3‘ |
| Gel retardation: |  |
| 0427nirB-up-fw | 5‘-GCCCTCCGGGCGGTATC-3‘ |
| 0427nirB-up-rev | 5‘-CATAGGGCGAGGCTAAGAACC-3‘ |
| up-0572-fw | 5‘-AAGTTACTGCGTGCCGAT-3‘ |
| up-0572-rev | 5‘-GGGTCTCTCCAGGGAGG-3‘ |
| up-0781-fw | 5‘-GTGCGGCGCGCGCGA-3‘ |
| up-0781-rev | 5‘-TGAGGGTCCTAATCCGTCCCTGAAC-3‘ |
| up-1052-fw | 5‘-GTGGCGCGGCAGGGC-3‘ |
| up-1052-rev | 5‘-TGGTTATTGCCTTCCCCCAG-3‘ |
| up-1084-fw | 5‘-CGCGGCGGTGCAGG-3‘ |
| up-1084-rev | 5‘-GTGTACTCGCAGAACTTCATCG-3‘ |
| up-1090-fw | 5‘-CAGGCCGGGCCTTCG-3‘ |
| up-1090-rev | 5‘-CCGGATGTTCCTTTCAGCTC-3‘ |
| up-1293-fw | 5‘-GGTGGCGGCGAGTTC-3‘ |
| up-1293-rev | 5‘-CGACTAAGAATCGGACAGA-3‘ |
| up-2184-fw | 5‘-CATGTGCCACCGTCGC-3‘ |
| up-2184-rev | 5‘-GGGTTTTCTCTCTGGCTAGG-3‘ |
| up-2425amtB-fw | 5‘-GACGTCGGGTTCGCGGCGCC-3‘ |
| up-2425amtBrev | 5‘-GTGTGAACCTCCTTGGGAAGTGCGCC-3‘ |
| up-2526-fw | 5‘-TAGCCGTGACCTCGTGACGAC-3‘ |
| up-2526-rev | 5‘-AACGGTGTGCTTCCTCCGC-3‘ |
| up-2748-fw | 5‘-CCCAGCTCGCCACTCTC-3‘ |
| up-2748-rev | 5‘-GGTCAGTGTGTACGCCGC-3‘ |
| up-2981-fw | 5‘-TTCGGTGTACCTGTGGAAG-3‘ |
| up-2981-rev | 5‘-ACGGTTGTCAGATCCCC-3‘ |
| 2982urtA-up-fw | 5‘-CATATCGACGCAGGCTAC-3‘ |
| 2982urtA-up-rev | 5‘-GAACAACCTTTCCCGATG-3‘ |
| up-3400-fw | 5‘-CGCGCGATCAGACCTTGC-3‘ |
| up-3400-rev | 5‘-AGGCTTTTCGCCGGCTGC-3‘ |
| up-4290-fw | 5‘-GGACCGGGACCGGATAG-3‘ |
| up-4290-rev | 5‘-TGAGTGTTCTCCTTTACTGGTAAC-3‘ |
| up-4294-fw | 5‘-CCGCGACGTCGACCG-3‘ |
| up-4294-rev | 5‘-GCTCCGCAGCGTATGCAC-3‘ |
| 4635amtA-up-fwNJ | 5‘-GACCACGCGCGTGCCG-3‘ |
| 4635amtA-up-revNJ | 5‘-AAAAACCTCCGCAGGGGAAG-3‘ |
| up-4638-fw | 5‘-CGCGGTGGTGGGCTG-3‘ |
| up-4638-rev | 5‘-GGGGTCACCACCTGCTG-3‘ |
| 5141narK-up-fw | 5‘-CGGGCGATCGACGATGG-3‘ |
| 5141narK-up-rev | 5‘-CCAGTCCAGGAAATCACCGG-3‘ |
| up-5734-fw | 5‘-GTGCTCGGCGTAGCTGC-3‘ |
| up-5734-rev | 5‘-GCGAGGCAGACTAGCGGC-3‘ |
| 5784glnR-ups-fw | 5‘-GGTGCCGACCGCCCCGACGA-3‘ |
| 5784glnR-ups-rev | 5‘-CAAGTCCTCCCGGCTCGTCAGGAATCTCT-3‘ |
| up-6258-fw | 5‘-ATGTCCATCTCGCGCGG-3‘ |
| up-6258-rev | 5‘-GTCCGCCAAGTTACCGGC-3‘ |
| 6259amt1-up-fw2 | 5‘-CGCTGGGACACCTCGGTGAC-3‘ |
| 6259amt1-up-rev | 5‘-CAAACATCTCCTCACGGTGGC-3‘ |
| up-6660-fw | 5‘-TGGCGATGGCGGATATC-3‘ |
| up-6660-rev | 5‘-GGCGTGCGATTCCGTTC-3‘ |
| 6734-up-fw | 5‘-GGGGTTGGCGATCAG-3‘ |
| 6734-up-rev | 5‘-TGCAGTATCACTACTTTCTTG-3‘ |
| up-6735-fw | 5‘-CGGTGCCGCGGTGGC-3‘ |
| up-6735-rev | 5‘-AGGCGCTCCTTCGGCG-3‘ |
| up-6816-fw | 5‘-CGGGGTCATCGGGCG-3‘ |
| up-6816-rev | 5‘-GGGCGGGTGTCCTCTG-3‘ |
| Strain construction: |  |
| Pupfw | 5’-CG**ATTTAAAT**GACGAACTCGACCTGAGC-3’ |
| Puprev | 5’-GA**TTAATTAA**GGTCTGCCCCGGCCTGCG-3’ |
| Pdownfw | 5’-GC**ACTAGT**CTGGCGGGCACCGTCGAC-3’ |
| Pdownrev | 5’-GC**GTTTAAAC**CGTGCGGTTGGTGAGCAT-3’ |
| amtRcom-fw | 5’-GCGC**TTAATTAA**ATGACGACCACCTCCGGC-3’ |
| amtRcom-rev | 5’-GCAG**ATTTAAAT**GGCACTCTAGCGGGCGGC-3’ |
| amtRe-fw | 5’-CGCGA**GGATCC**ATGACGACCACCTCCGGCC-3’ |
| amtRe-rev | 5’-CGC**AAGCTT**AGTGATGGTGATGGTGATGGGGCGGTCGCACA  CCGAGCC-3’ |
| EMSA: |  |
| msmeg_2184pfw | 5’-GCCGACCACGCATGTGCCAC-3’ |
| msmeg_2184prev | 5’-CGGCGAACGACTCGAACCGG-3’ |
| msmeg_4301pfw | 5’-ATGAGGGCGAGACTGTGG-3’ |
| msmeg_4301prev | 5’-GTCTGGGATGGCGATGTT-3’ |
